# New *γ*-Halo-*δ*-lactones and *δ*-Hydroxy-*γ*-lactones with Strong Cytotoxic Activity

**DOI:** 10.3390/molecules24101875

**Published:** 2019-05-15

**Authors:** Angelika Kamizela, Barbara Gawdzik, Mariusz Urbaniak, Łukasz Lechowicz, Agata Białońska, Sylwia Ewa Kutniewska, Weronika Gonciarz, Magdalena Chmiela

**Affiliations:** 1 Institute of Chemistry, Jan Kochanowski University, Świętokrzyska 15 G, 25-406 Kielce, Poland; b.gawdzik@ujk.edu.pl (B.G.); mariusz.urbaniak@ujk.edu.pl (M.U.); 2 Institute of Biology, Jan Kochanowski University, Świętokrzyska 15 G, 25-406 Kielce, Poland; llechowicz@ujk.edu.pl; 3 Department of Chemistry, University of Wroclaw, F. Joliot-Curie 14, 50-383 Wrocław, Poland; agata.bialonska@chem.uni.wroc.pl; 4 Department of Chemistry, University of Warsaw, Żwirki i Wigury 101, 02-089 Warszawa, Poland; sekutyla@gmail.com; 5 Department of Immunology and Infectious Biology, University of Lodz, Banacha 12/16, 90-237 Łódź, Poland; weronika.gonciarz@biol.uni.lodz.pl (W.G.); magdalena.chmiela@biol.uni.lodz.pl (M.C.)

**Keywords:** halolactones, translactonization, hydroxylactones, anti-cancer properties, bactericidal properties

## Abstract

This paper presents the synthesis of γ-halo-δ-lactones, δ-iodo-γ-lactones and δ-hydroxy-γ-lactones from readily available organic substrates such as *trans*-crotonaldehyde and aryl bromides. Crystal structure analysis was carried out for lactones that were obtained in crystalline form. All halo-δ-lactones and δ-hydroxy-γ-lactones were highly cytotoxic against gastric cancer AGS cells with $IC_{50}$ values in the range of 0.0006–0.0044 mM. Some lactones showed high bactericidal activity against *E. coli* ATCC 8739 and *S. aureus* ATCC 65389, which reduced the number of CFU/mL by 70–83% and 87% respectively.

## 1. Introduction

Lactones are compounds with unusual biological activities. The natural source of lactones are plants [1,2] but they are also found in microorganisms [3] and animals [4]. They exhibit, among others, anti-cancer activity [5,6], cytotoxic [7,8,9], bacterio- [10,11,12] and fungistatic [13,14], antiviral [15,16] and deterrent properties [17,18]. This biological potential is widely used in medicine and agriculture. Lactones also have sensory properties [19] used in the cosmetics and food industries but they can also be toxic, such as mycotoxins produced by mold fungi [20]. The properties and application possibilities of lactones are they reason they are still intensively studied by many groups. New natural derivatives are still isolated, their properties are examined and new synthetic analogs with desirable properties are sought. This interesting group of compounds also attracted our attention. Thus far, we have developed a synthesis of β-ayl-δ-iodo-γ-lactones [21], aryl-δ-hydroxy-γ-lactones [22], and trans-γ-halo-δ-lactones [23], and tested their cytotoxic and bactericidal activities. Here, we present stereoselective substrate-controlled synthesis of new γ-halo-δ-lactones. Some of the obtained iodo-δ-lactones can easily undergo translactonization reactions leading to δ-hydroxy-γ-lactones with high yields. Their biological activities were also evaluated: antimicrobial activity against *Escherichia coli* strain ATCC 8739 and *Staphylococcus aureus* strain ATCC 65389, and cytotoxicity against L929 cell lines (mouse fibroblasts) and human gastric cancer cell line AGS.

## 2. Results and Discussion

### 2.1. Synthesis

**Figure 1:**
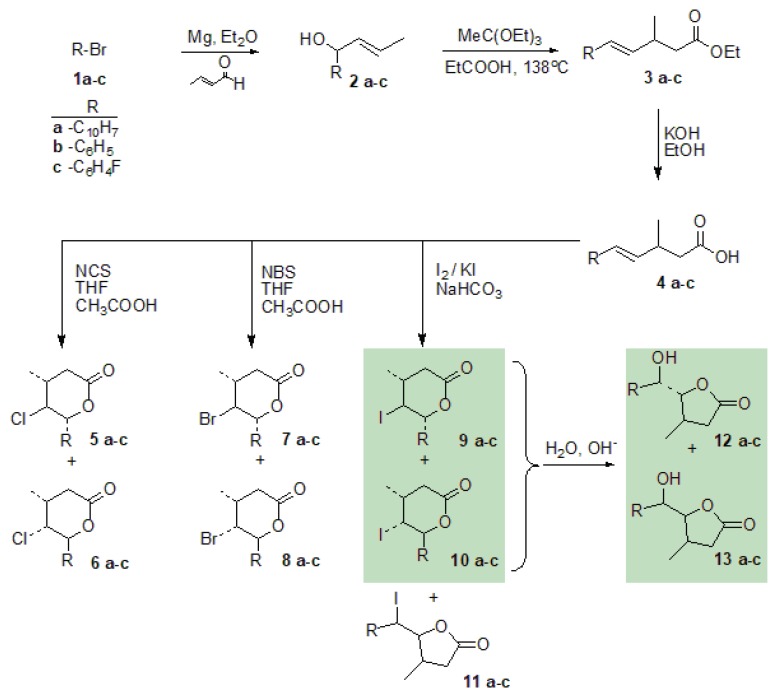
A four-step synthesis of γ-halo-δ-lactones and δ-hydroxy-γ-lactones.

Halolactones were obtained as a result of the four-step reaction [23] shown in Figure 1. The first step of the synthesis was a Grignard reaction between selected aryl bromides **1a**–**c** and trans-crotonaldehyde, which gave us unsaturated allyl alcohols **2a**–**c** with yields 48–62% [24]. The Johnson–Claisen rearrangement [25] of these alcohols with triethyl orthoacetate and catalytic amount of propionic acid obtained γ,δ-unsaturated ethyl esters **3a**–**c** with yields 49–63%. In the third step, the esters were subjected to alkaline hydrolysis in ethanol to obtain γ,δ-unsaturated carboxylic acids **4a**–**c**. Thus obtained substrates, with suitably substituted double bonds, enforced selectivity of halolactonization in the last step of the synthesis. The yields and products distribution are shown in Table 1. Only two of these compounds have been described thus far in the literature [26].

**Figure 2:**
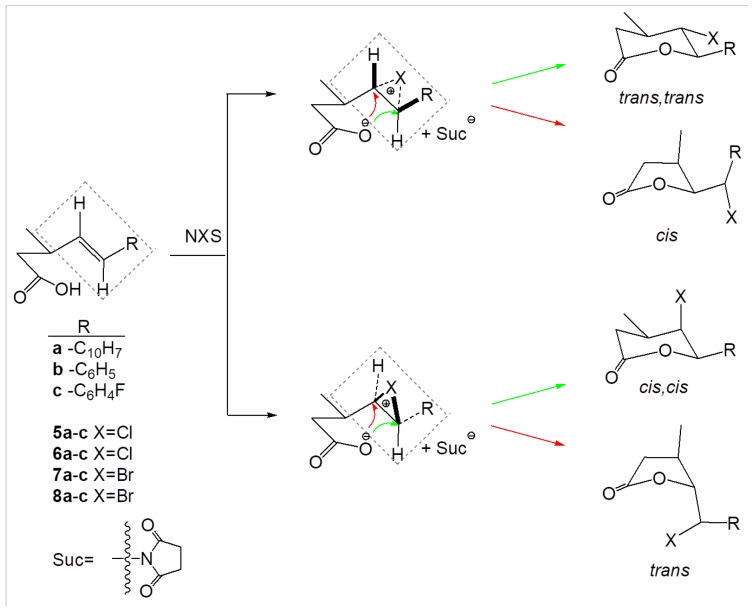
Mechanisms of chloro- and bromolactonization of γ,δ-unsaturated carboxylic acids with NCS and NBS.

According to the literature [27,28], halolactonization of γ,δ-unsaturated carboxylic acids, carried out with *N*-chlorosuccinimide (NCS) or N-bromosuccinimide (NBS), leads to a mixture of γ- and δ-lactones, with an excess of 6-*endo* cyclization products. The regioselectivity of the reaction is influenced by steric hindrance at carbon atoms adjacent to the double bond. In the case of acids with substituents at the C-3, nucleophilic attack at the γ-position is hindered and the amount of products formed by 5-*exo* cyclization decreases. This is also confirmed by our results. The chlorolactonization of unsaturated carboxylic acids **4a**–**c** with N-chlorosuccinimide (NCS) in THF, only proceeded through a 6-*endo* cyclization mechanism and gave new γ-halo-δ-lactones **5a**–**c** and **6a**–**c**. The analysis of post-reaction mixtures showed that in each case only isomers *trans,trans* and *cis,trans* were formed (Figure 2). The thermodynamically more stable isomers *trans,trans* were always present in large excess, up to 80% (Table 1 and Table 2). An analogous synthesis using γ,δ-unsaturated carboxylic acids with a methyl group in the β-position led to a mixture of products. Lactonization of unsaturated carboxylic acids with two methyl groups in the β-position have only one product, *trans*-γ-chloro-δ-lactone [23].

Bromolactones **7a**–**c** and **8a**–**c** were obtained by reacting **4a**–**c** with N-bromosuccinimide (NBS) in THF with a yield of 72–78%. Mechanisms for lactonization of γ,δ-unsaturated carboxylic acids with NCS and NBS are similar [27] (Figure 2) and lead to the same products of 6-*endo* cyclization. The analysis of post-reaction mixtures showed that in bromolactonization of unsaturated carboxylic acids **4a**–**c** in each case only isomers *trans,trans* and *cis,trans* were formed. The yield of *trans,trans*-γ-bromo-δ-lactones was 76–82% and yield of *cis,trans*-γ-bromo-δ-lactones was only 18–24%. The previously described reactions of γ,δ-unsaturated carboxylic acids with $I_{2}/KI$ in saturated $NaHCO_{3}$ solution, carried out under kinetic control, resulted in mixtures of γ- and δ-iodolactones, in which the products formed by 5-*exo* cyclization predominated. The regioselectivity-determining step for the reaction is the attack of the negatively charged oxygen atom of the carboxyl group on the iodine-double bond complex. The presence of substituents in the β-position of unsaturated carboxylic acids hindered nucleophilic attack at the C-4 position. This limited the formation of five-membered lactones. The effect of steric hindrance at the C-6 negatively influenced the formation of δ lactones and favored γ-lactones [27].

**Figure 3:**
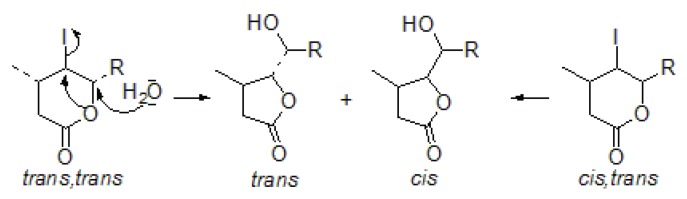
The translactonization mechanism of γ-iodo-δ-lactones.

Iodolactonization of **4a**–**c** acids was carried out with $I_{2}/KI$ and $NaHCO_{3}$ in a water/diethyl ether mixture [29]. Analysis of the reaction mixtures unexpectedly showed the formation of two new isomers of δ-hydroxy-γ-lactone **12a** and **13a**. The yield of reactions was in the range of 69–85%. The reaction conditions prevented the direct synthesis of these compounds because the reaction was carried out in an alkaline solution, whereas hydroxylactonization requires an acidic environment [22]. *Cis*-isomers are more thermodynamically stable than *trans*-isomers and predominantly formed in this reaction. The excess of *trans*-isomer is usually observed in the synthesis of γ-lactones. However, the reaction initially gave δ-iodo-γ-lactones, which, through a translactonization reaction, were transformed into the final products–hydroxylactones **12a** and **13a**. This was confirmed by observations of purified isomers *trans,trans* and *cis,trans* γ-iodo-δ-lactones **9b** and **10b**, and **9c** and **10c**. These isomers underwent rapid decomposition with release of iodine. The analysis of residues showed that pure isomers *trans,trans* or *cis,trans* transformed into an equimolar mixture of isomers *trans* and *cis* hydroxylactones (Figure 3). These results indicate that the translactonization of γ-iodo-δ-lactones involved elimination of the iodine, which was a good leaving group and rearrangement of the six-membered lactone ring into the five-membered one, according to the SN1 mechanism. The carbocation was stabilized by the electron lone pairs of the oxygen atom present in the water molecule. The preparation of hydroxylactones from iodolactones has been described thus far only for biotransformation experiments. The synthesis described here allows obtaining these compounds without microorganisms.

Iodolactonization of γ,δ-unsaturated carboxylic acids **4b** and **4c** also led to results different from previous studies (Table 1). In both reactions, five products with the lactone ring were identified. The main products were isomers *trans,trans*-γ-iodo-δ-lactones **9b** (yield 42%) and **9c** (yield 37%). Isomers *cis,trans*-γ-iodo-δ-lactones **10b,c** were formed with yields of 30% and 33% and isomers *cis*-δ-iodo-γ-lactones **11b,c** only with yields of 10% and 8%. This result was new and unexpected, because iodolactonization of γ,δ-unsaturated carboxylic acid usually gives a mixture of γ-lactones as the main product and only a trace of δ-lactones [27]. This reaction also produced δ-hydroxy-γ-lactones. Isomers trans **12b,c** were formed with yields of 5% and 7% and isomers *cis* **13b,c** with yields of 14% and 18%.

### 2.2. The Structural Analysis of Compounds

**Figure 4:**
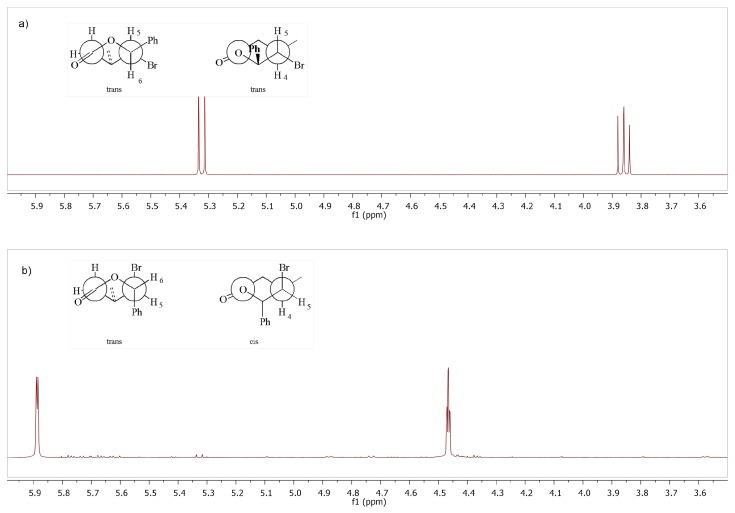
The comparison of the NMR signals for H-6 and H-5 protons of: (**a**) *trans,trans*-γ-bromo-δ-lactone **7b** and (**b**) *cis,trans*-γ-bromo-δ-lactone **8b**.

Spectroscopic and spectrometric analyses were performed for all obtained compounds. The X-ray structural analysis was carried out for the solid products of lactonization. The ${}^{1}H$ NMR spectra of analogous isomers of γ-halo-δ-lactones are similar to each other in most respects. In *cis,trans*-γ-bromo-δ-lactone **8b**, the H-6 proton was observed at 5.89 ppm as a doublet with coupling constants 2.5 Hz (Figure 4). The signal of proton H-5 appears at 4.46 ppm as a triplet due to coupling with protons H-4 and H-6 and the coupling constant 2.5 Hz. According to Karplus, the coupling constant of vicinal protons depends on the dihedral angle between them. The equatorial-equatorial and axial-equatorial coupling constants are in the range 1–5 Hz. The axial-axial coupling constant is normally large but these magnitudes are decreased (9–10 Hz) by the antiperiplanar arrangement of the hydrogens and oxygen atom of the carbonyl group [30,31].

**Figure 5:**
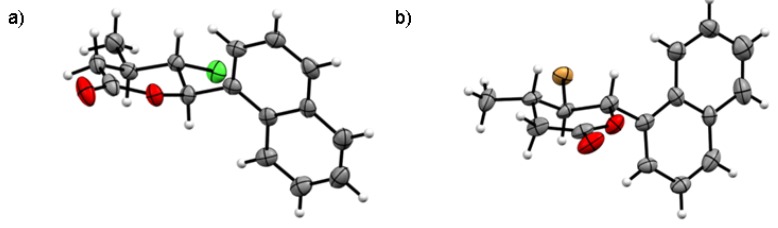
The crystalline structure of: (**a**) *trans,trans*-γ-chloro-δ-lactone **5a**; and (**b**) *trans,trans*-γ-bromo-δ-lactone **7a**.

The magnitudes of these coupling constants indicate that the protons H-5 and H-6 are in aquatorial positions and proton H-5 is in axial position. This causes that in *cis,trans*-γ-chloro-δ-lactones **8b** Br and Ph substituents are on opposite sides of the lactone ring, in the axial position at C-5 and C-6 and CH${}_{3}$ substituent is in equatorial position at C-4 carbon atom. In the case of *trans,trans* isomer **7b**, coupling constant between protons H-4, H-5 and H-6 is much larger. The signal of protons H-6 observed at 5.32 ppm as a doublet with coupling constant *J*${}_{H5/H6}$ = 10.3 Hz. The triplet observed at 3.86 ppm for H-5 proton have a coupling constant 10.2 Hz. This means that all the substituents are in the equatorial position, alternately above and below the lactone ring plane. The pyranose chair conformations with substituents in the equitorial position are more stable than in the axial position due to torsional strain and 1,3-diaxial interactions. This is reflected in the composition of the reaction mixture where *trans* isomers are dominant. The structure of the *trans,trans*-γ-halo-δ-lactones also have been confirmed by single crystal X-ray diffraction analysis. Figure 5a shows the crystalline structure of *trans,trans*-γ-chloro-δ-lactone **5a** [32]. The dihedral angles C1${}^{\prime }$-C6-C5-Cl and C7-C4-C5-Cl being $67.01^{\circ }$ and $57.24^{\circ }$ respectively. Thus, the aryl substituent at C-6, chlorine atom at C-5 and methyl group at C4 are in the equatorial positions in *gauche,gauche* arrangement. Hydrogen atoms H-4, H-5 and H-6 occupy the axial position with the dihedral angles between H-4 and H-5 and between H-5 and H-6 equal to $175.72^{\circ }$ and $172.61^{\circ }$, respectively. X-ray analysis of *trans,trans*-γ-chloro- and *trans,trans*-γ-bromo-δ-lactones showed that the structure of the lactone rings of the same isomers is analogous. Figure 5b shows the crystalline structure of *trans,trans*-γ-bromo-δ-lactone **7a** [33].

**Figure 6:**
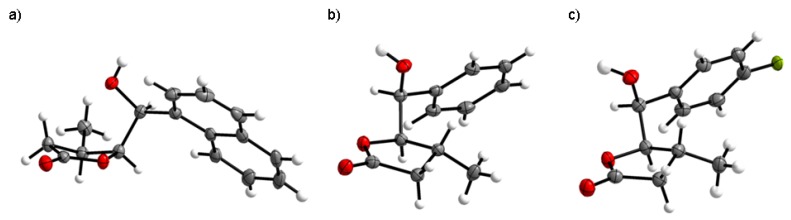
The crystalline structure of: (**a**) *cis*-δ-hydroxy-γ-lactone **1**3a; (**b**) *trans*-δ-hydroxy-γ-lactone **1**2b; and (**c**) *trans*-δ-hydroxy-γ-lactone **1**2c.

The crystalline structure of γ-hydroxylactone **13a** [34], obtained as a result of iodolactonization of acid **4a** in 90% yield, is shown in Figure 6a. The dihedral angles C7-C4-C5-C6 and H4-C4-C5-H5 are $33.36^{\circ }$ and $29.31^{\circ }$, respectively. Thus, the substituents at C-4 and C-5 are in syn-periplanar orientation, on the same side of the lactone ring, forming the *cis* isomer.

**Figure 7:**
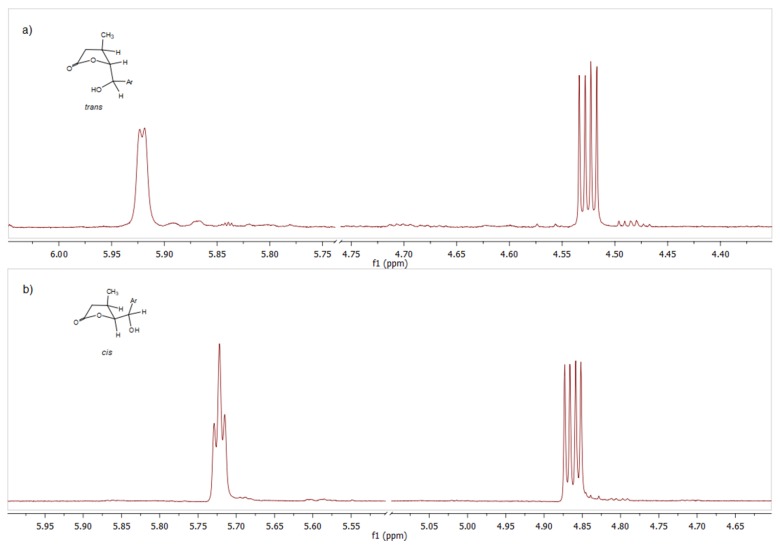
The comparison of the NMR signals for H-5 and H-6 protons of: (**a**) *trans*-δ-hydroxy-γ-lactone **12b**; and (**b**) *cis*-δ-hydroxy-γ-lactone **13a**.

The structure of γ-hydroxylactones was also confirmed by NMR spectroscopic analysis. In the ${}^{1}H$ NMR spectra of *cis*-γ-hydroxylactone **13a**, the signal of proton H-5 appears at δ = 4.86 ppm as a double doublet, due to coupling with protons H-4 and H-6 (*J*${}_{H4/H5}$ = 7.2 Hz, *J*${}_{H5/H6}$ = 3.4 Hz). The analogous coupling constants for *trans*-γ-hydroxylactone **12a** are smaller and equal to 5.5 Hz and 2.8 Hz, respectively (Figure 7). This means that the substituents are in the equatorial position in *trans*-arrangement.

The structure of the lactone rings was also confirmed by ${}^{13}C$ NMR and IR spectroscopy. The carbon signals at δ: 68.97 (**13a**) and 72.13 ppm (**12a**) are characteristic of carbon atoms bonded to the hydroxyl group. The typical chemical shift values for the ester carbon atoms in γ-lactones also have signals at 177.11 ppm (**13a**) and 175.98 ppm (**12a**). In the IR spectrum, the stretching bands of the C = O group appeared at 1747 cm${}^{-1}$ (**13a**) and 1774 cm${}^{-1}$ (**12a**). Spectroscopic analysis showed a similar structure of lactones **13b,c** and **12b,c**. The crystalline structures of γ-hydroxylactones **12b** [35] and **12c** [36] are shown in Figure 6b,c. The dihedral angles C7-C4-C5-C6 and H4-C4-C5-H5, being $96.54^{\circ }$ and $135.80^{\circ }$ (**12b**), $97.08^{\circ }$ and $147.93^{\circ }$ (**12c**), respectively, are characteristic of *antiperiplanar* conformers and *trans* configuration of the lactone rings.

The iodolactonization of γ,δ-unsaturated carboxylic acids **4b,c** also gave δ-iodo-γ-lactones **11b** and **11c** in low yields (7% and 8%, respectively). Their structure was determined by spectroscopic methods. In the IR spectra, the absorption bands of the C = O group were observed at 1765 cm${}^{-1}$ (**11b**) and 1760 cm${}^{-1}$ (**11c**). This is the typical range for γ-lactones. The analysis of NMR spectra showed the signal of the carbonyl carbon at δ 176.00 ppm (**11b**) and 176.11 ppm (**11c**). The signals observed at δ: 28.38 ppm (**11b**) and 28.45 ppm (**11c**) were assigned to carbons connected to the iodine. The signal of proton H-5 appears as a double doublet, due to coupling with protons H-4 and H-6 (*J*${}_{H4/H5}$ = 11.2 Hz, *J*${}_{H5/H6}$ = 4.5 Hz **11b,c**). This indicates the syn-periplanar orientation of the protons H-4 and H-5, and *cis* configuration of γ-lactones.

### 2.3. Bactericidal Properties

The results of CFU counting method are shown in Table 3. The lactones **6c**, **9c** and **10c** showed the highest bactericidal activity against *E. coli* ATCC 8739 (70–78% less CFU/mL). The other lactones exhibited moderate bactericidal properties. The highest bactericidal activity against *S. aureus* ATCC 65389 was found for lactone **5a** which reduced the number of CFU/mL by 87%. The bactericidal properties against tested bacterial strain also showed lactones **6b**, **8c** and **7a** (78–83% less CFU/mL). Lactones **9a** and **12a** did not show bactericidal activity against *S. aureus* ATCC 65389.

#### The 3-(4,5-Dimethylthiazol-2-Yl)-2,5-diphenyltetrazolium Bromide ( Mtt) Reduction Effectiveness

All lactones inhibited metabolic activity of the cell lines in the concentration range of 0.1–50 μg/mL. The ability of cells to reduce MTT to formazan decreased by 37–93% (Table 4 and Table 5). The cytotoxicity of tested lactones against normal eukaryotic cells may exclude their use as antimicrobial agents in medicine but does not exclude their use in anti-cancer drugs. Our results showed that lactones **10b**, **10c** and **6b** exhibit the lowest $IC_{50}$ values against L929 cells. All halo-δ-lactones and δ-hydroxy-γ-lactones were highly cytotoxic against gastric cancer AGS cells with $IC_{50}$ values in the range of 0.0006–0.0044 μM. These values are much lower than $IC_{50}$ = 0.025 μM of doxorubicin, the reference anticancer drug [37]. The differences between $IC_{50}$ values for AGS and L929 cell line were evaluated by non-parametric Mann–Whitney *U* test. Statistical significance (*p* ≤ 0.05) was observed for all tested lactones except **6b** (*p* = 0.1).

Such cytostatic activity of lactones was reported by various research groups. Bai et al. showed the cytotoxic effects of sesquiterpene lactones from *Inula britannica* against four human cancer cell lines: COLO 205, HT 29, HL-60 and AGS [38]. The high cytotoxicity of halo lactones with β-phenyl-γ-lactone or β-phenyl-δ-lactone framework against cancer lines Jurkat (human leukaemia) and D17 (canine osteosarcoma) was reported by Mazur et al. [28,39,40].

## 3. Materials and Methods

All used chemicals were purchased from Sigma-Aldrich and Fluka and used without further purification. The NMR spectra were obtained in CDCl${}_{3}$ on a Bruker Avance DRX 500 MHz. FTIR spectra were recorded using an attenuated total reflection technique on a Perkin Elmer Spectrum 400 spectrometer. High-resolution electrospray ionization mass spectra (HR-ESI-MS) were acquired on a Bruker micrOTOF-Q II. Gas chromatography was performed using a Thermo Scientific-Trace 1310 chromatograph equipped with a TG-5HT column (30 m × 0.25 mm). Melting points were determined with a Boetius micro melting point apparatus and are uncorrected. The refractive index was determined with an Abbe refractometer (Atago RX-7000 CX). TLC analysis was performed on silica gel F254 plates (Merck). Column chromatography was performed on silica gel 60 (230–400 mesh, Merck) using mixtures of hexane, ethyl acetate and acetone as eluents. The crystal structure was determined by single-crystal X-ray diffraction (Xcalibur, Sapphire 2 CCD detector).

### 3.1. X-ray Study

**Figure 8:**
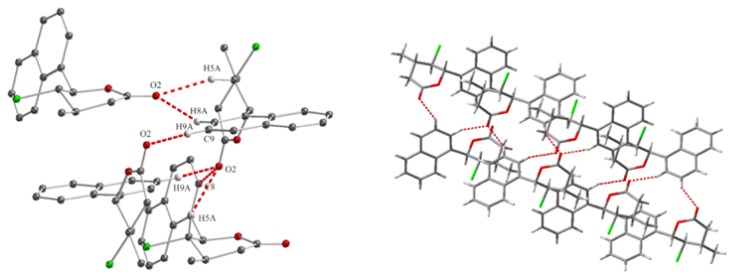
The arrangement of **5a** in the unit cell box.

**Figure 9:**
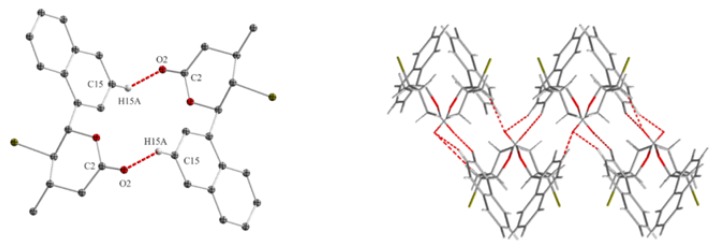
The arrangement of **7a** in the unit cell box.

**Figure 10:**
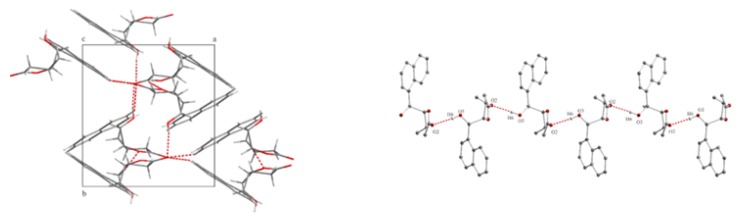
The arrangement of **13a** in the unit cell box.

**Figure 11:**
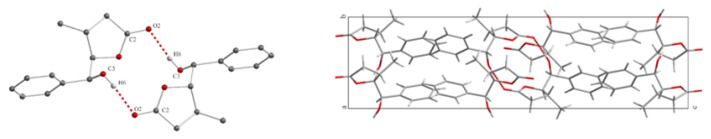
The arrangement of **12b** in the unit cell box.

**Figure 12:**
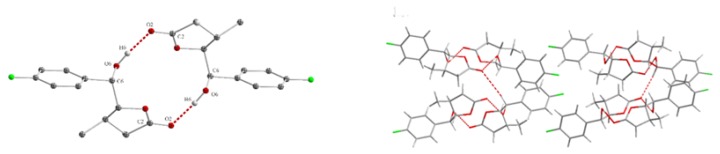
The arrangement of **12c** in the unit cell box.

The single crystal X-ray diffraction for lactones was carried out on a Rigaku Oxford Diffraction SuperNova diffractometer equipped with a micro focus Cu X-ray source. The crystals were maintained at 100 K by using an Oxford Cryosystems nitrogen gas-flow device. Data collection strategies were optimized using CrysAlisPro software package [41]. The crystal structures of lactones were solved using the charge-flipping method implemented in SUPERFLIP and refined with the JANA package [42]. The crystal data, data collection, and refinement parameters for lactones are given in Table 6. Crystallographic data, as CIF files, have been deposited with the Cambridge Crystallographic Data Centre. The solid-state structure of lactone was determined by single-crystal X-ray diffraction. The lactones **5a** (Figure 8), **7a** (Figure 9), and **13a** (Figure 10) crystallized in the centrosymmetric space group $P2_{1/c}$ while lactones **12b** (Figure 11) and **12c** (Figure 12) in the orthorhombic space group Pbca. Only one molecule is present in the asymmetric unit. Experimental details of the crystallographic analysis are given in Table 5.

### 3.2. Claisen Rearrangement

The γ,δ-unsaturated ethyl esters **3a**–**c** were obtained by Claisen rearrangement [25]. A mixture of triethyl orthoacetate (0.15 mol), ethanol (0.02 mol) and a catalytic amount (one drop) of propionic acid was heated at 138 ${}^{\circ }$C for 5 h while distilling off alcohol. When the reaction was completed (TLC), the excess of triethyl orthoacetate was removed by evaporation. The crude product was purified by column chromatography on silica gel using a mixture of ethyl acetate and hexane (1:80). Spectral data are given below.

Ethyl 3-methyl-5-(naphthalen-1-yl)pent-4-enoate (**3a**): The product was obtained as a yellow oil with a yield of 62.75%; $n_{D}^{20}$ = 1.5641; $R_{f}$ = 0.38 (EtOAc:hexane 1:20); ${}^{1}$H NMR (CDCl${}_{3}$, 600 MHz), δ [ppm]: 1.28 (d, 3H, *J* = 6.8 Hz, $CH(CH_{3}$)), 1.29 (t, 3H, *J* = 7.1 Hz, OCH${}_{2}$CH${}_{3}$), 2.48 (dd, 1H, $J_{1}$ = 14.7 Hz, $J_{2}$ = 7.1 Hz, CH_a_H_b_), 2.55 (dd, 1H, $J_{1}$ = 14.7 Hz, $J_{2}$ = 7.6 Hz, CH_a_H_b_), 2.99–3.10 (m, 1H, $CH(CH_{3}$)), 4.12 (dd, 2H, $J_{1}$ = 7.1 Hz, $J_{2}$ = 1.7 Hz, $CH_{2}CH_{3}$), 6.19 (dd, 1H, $J_{1}$ = 15.6 Hz, $J_{2}$ = 7.6 Hz, CH = CH), 7.19 (d, 1H, *J* = 15.6 Hz, CH = CH), 7.45–7.60 (m, 4H, HAr), 7.79 (d, 1H, *J* = 8.2 Hz, HAr), 7.87 (d, 1H, *J* = 7.7 Hz, HAr), 8.13 (d, 1H, *J* = 8.2 Hz, HAr); ${}^{13}$C NMR (151 MHz, CDCl${}_{3}$), δ [ppm]: 14.34 (OCH${}_{2}$CH${}_{3}$), 20.39 ($CH(CH_{3}$)), 34.57 (CH(CH${}_{3}{)}_{2}$), 41.93 (CH${}_{2}$), 60.38 (OCH${}_{2}$CH${}_{3}$), 125.90 (CH = CH), 133.62 (CH = CH), 172.50 (C = O), CAr: 123.74, 123.92, 125.65, 125.71, 125.90, 126.23, 127.55, 128.50, 131.21, 135.33, 137.66; IR (cm${}^{-1}$): 1727, 1589, 1507, 1455, 1392, 1365, 1345, 1268, 1239, 1164, 1094, 1028, 968, 861, 791, 775, 732; HR-MS (ESI-TOF) calculated for C${}_{18}$H${}_{20}$O${}_{2}$, *m*/*z* [M + Na]${}^{+}$: 291.136092; experimental value: 291.137301.

Eethyl 3-methyl-5-phenylpent-4-enoate (**3b**): The product was obtained as a yellow oil with a yield of 59.26%; $n_{D}^{20}$ = 1.5201; $R_{f}$ = 0.37 (EtOAc:hexane 1:20); ${}^{1}$H NMR (CDCl${}_{3}$, 500 MHz), δ [ppm]: 1.15 (d, 3H, *J* = 6.8 Hz, CH(CH${}_{3}$)), 1.23 (t, 3H, *J* = 7.1 Hz, OCH${}_{2}$CH${}_{3}$), 2.35 (dd, 1H, $J_{1}$ = 14.8 Hz, $J_{2}$ = 7.3 Hz, CH_a_H_b_), 2.42 (dd, 1H, $J_{1}$ = 14.8 Hz, $J_{2}$ = 7.3 Hz, CH_a_H_b_), 2.80–2.92 (m, 1H, $CH(CH_{3}$)), 4.12 (q, 2H, *J* = 7.1 Hz, OCH${}_{2}$CH${}_{3}$), 6.14 (dd, 1H, $J_{1}$ = 15.9 Hz, $J_{2}$ = 7.6 Hz, CH = CH), 6.40 (d, 1H, *J* = 15.9 Hz, CH = CH), 7.17–7.39 (m, 5H, HAr); ${}^{13}$C NMR (125 MHz, CDCl${}_{3}$), δ [ppm]: 13.82 (OCH${}_{2}$CH${}_{3}$), 19.74 ($CH(CH_{3}$)), 33.62 ($C{\left(CH_{3}\right)}_{2}$), 41.30 (CH${}_{2}$), 59.79 (OCH${}_{2}$CH${}_{3}$), 125.63 (CH = CH), 133.79 (CH = CH), 171.91 (C = O), CAr: 126.08, 126.63, 128.01, 136.95; IR (cm${}^{-1}$): 1731, 1597, 1494, 1450, 1368, 1348, 1298, 1285, 1239, 1162, 1100, 1079, 1071, 1030, 964, 929, 909, 842, 747, 690; HR-MS (ESI-TOF) calculated for C${}_{14}$H${}_{18}$O${}_{2}$, *m*/*z* [M + Na]${}^{+}$: 241.120443; experimental value: 241.121304.

Ethyl 5-(4-fluorophenyl)-3-methylpent-4-enoate (**3c**): The product was obtained as a yellow oil with a yield of 48.50%; $n_{D}^{20}$ = 1.5201, $R_{f}$ = 0.38 (EtOAc:hexane 1:20); ${}^{1}$H NMR (CDCl${}_{3}$, 500 MHz), δ [ppm]: 1.15 (d, 3H, *J* = 6.8 Hz, CH(CH${}_{3}$)), 1.23 (t, 3H, *J* = 7.1 Hz, OCH${}_{2}$CH${}_{3}$), 2.35 (dd, 1H, $J_{1}$ = 14.8 Hz, $J_{2}$ = 7.2 Hz, CH_a_H_b_), 2.41 (dd, 1H, $J_{1}$ = 14.8 Hz, $J_{2}$ = 7.4 Hz, CH_a_H_b_), 2.78–32.92 (m, 1H, CH(CH${}_{3}$)), 4.13 (q, 2H, *J* = 7.1 Hz, OCH${}_{2}$CH${}_{3}$)), 6.05 (dd, 1H, $J_{1}$ = 15.9 Hz, $J_{2}$ = 7.6 Hz, CH = CH), 6.36 (d, 1H, *J* = 15.9 Hz, CH = CH), 6.98 (t, 2H, *J* = 8.7 Hz, HAr), 7.24–37.32 (m, 2H, HAr); ${}^{13}$C NMR (125 MHz, CDCl${}_{3}$), δ [ppm]: 14.27 (OCH${}_{2}$CH${}_{3}$), 20.19 (CH(CH${}_{3}$)), 34.04 (CH(CH${}_{3}$)), 41.74 (CH${}_{2}$), 60.26 (OCH${}_{2}$CH${}_{3}$), 127.68 (CH = CH), 133.99 (CH = CH), 172.32 (C = O), CAr: 115.32, 127.53, 133.55, 162.03; IR (cm${}^{-1}$): 1732, 1603, 1506, 1459, 1415, 1370, 1348, 1298, 1283, 1225, 1207, 1174, 1156, 1095, 1079, 1028, 970, 935, 851, 814, 772; HR-MS (ESI-TOF) calculated for C${}_{14}$H${}_{17}$FO${}_{2}$, *m*/*z* [M + Na]${}^{+}$: 259.111021; experimental value: 259.111099.

### 3.3. Basic Hydrolysis of Esters

The γ,δ-unsaturated carboxylic acids were obtained by alkaline hydrolysis of esters **3a**–**c**. The reaction occurred in ethanolic KOH under reflux conditions (2 h). The excess of ethanol was evaporated and the residue was acidified with 0.1 M hydrochloric acid. The product was extracted with diethyl ether and dried over anhydrous magnesium sulfate. Spectral data are given below.

3-Methyl-5-(naphthalen-1-yl)pent-4-enoic acid (**4a**): The product was obtained as a colorless oil with a yield of 98%; $n_{D}^{20}$ = 1.5940; $R_{f}$ = 0.16 (EtOAc:hexane 1:5); ${}^{1}$H NMR (CDCl${}_{3}$, 500 MHz), δ [ppm]: 1.23 (d, 3H, *J* = 6.8 Hz, CH(CH${}_{3}$)), 2.46 (dd, 1H, $J_{1}$ = 15.2 Hz, $J_{2}$ = 7.1 Hz, CH_a_H_b_), 2.54 (dd, 1H, $J_{1}$ = 15.2 Hz, $J_{2}$ = 7.4 Hz, CH_a_H_b_), 2.92–3.03 (m, 1H, CH(CH${}_{3}$)), 6.13 (dd, 1H, $J_{1}$ = 15.6 Hz, $J_{2}$ = 7.5 Hz, CH = CH), 7.15 (d, 1H, *J* = 15.6 Hz, CH = CH), 7.53–7.37 (m, 5H, HAr), 7.72 (d, 1H, *J* = 8.2 Hz, HAr), 7.81 (d, 1H, *J* = 8.7 Hz, HAr), 8.06 (d, 1H, *J* = 8.5 Hz, HAr), 9.28 (s, 1H, COOH); ${}^{13}$C NMR (125 MHz, CDCl${}_{3}$), δ [ppm]: 20.25 (CH(CH${}_{3}$)), 34.14 (CH(CH${}_{3}$)), 41.50 (CH${}_{2}$), 125.86 (CH = CH), 137.16 (CH = CH), 178.47 (C = O), CAr: 123.70, 123.84, 125.57, 125.65, 126.46, 127.54, 128.41, 131.13, 133.52, 135.15; IR (cm${}^{-1}$): 2600, 1704, 1591, 1507, 1451, 1406, 1394, 1374, 1288, 1230, 1170, 1140, 1089, 1069, 1013, 965, 911, 857, 791, 773, 730; HR-MS (ESI-TOF) calculated for C${}_{16}$H${}_{16}$O${}_{2}$, *m*/*z* [M + Na]${}^{+}$: 263.104794; experimental value: 263.105649.

3-methyl-5-phenylpent-4-enoic acid (**4b**): The product was obtained as a colorless oil with a yield of 96.5%; $n_{D}^{20}$ = 1.5434; $R_{f}$ = 0.15 (EtOAc:hexane 1:5); ${}^{1}$H NMR (CDCl${}_{3}$, 500 MHz), δ [ppm]: 1.17 (d, 3H, *J* = 6.8 Hz, CH(CH${}_{3}$)), 2.39 (dd, 1H, $J_{1}$ = 15.3 Hz, $J_{2}$ = 7.3 Hz, CH_a_H_b_), 2.48 (dd, 1H, $J_{1}$ = 15.3 Hz, $J_{2}$ = 7.2 Hz, CH_a_H_b_), 2.80–2.91 (m, 1H, CH(CH${}_{3}$)), 6.14 (dd, 1H, $J_{1}$ = 15.9 Hz, $J_{2}$ = 7.5 Hz, CH = CH), 6.41 (d, 1H, *J* = 15.9 Hz, CH = CH), 7.16–7.37 (m, 5H, HAr), 10.14 (s, 1H, COOH); ${}^{13}$C NMR (125 MHz, CDCl${}_{3}$), δ [ppm]: 20.17 (CH(CH${}_{3}$)), 33.66 (CH(CH${}_{3}$)), 41.39 (CH${}_{2}$), 127.09 (CH = CH), 133.84 (CH = CH), 178.68 (C = O), CAr: 126.14, 128.50, 129.05, 137.30; IR (cm${}^{-1}$): 2630, 1702, 1598, 1494, 1446, 1408, 1374, 1347, 1288, 1250, 1218, 1173, 1144, 1096, 1074, 1024, 965, 931, 909, 859, 841, 746, 689, 664; HR-MS (ESI-TOF) calculated for C${}_{12}$H${}_{14}$O${}_{2}$, *m*/*z* [M + Na]${}^{+}$: 213.089144; experimental value: 213.088930.

5-(4-fluorophenyl)-3-methylpent-4-enoic acid (**4c**): The product was obtained as a colorless oil with a yield of 98.0%; $n_{D}^{20}$ = 1.5205, $R_{f}$ = 0.16 (EtOAc:hexane, 1:5); ${}^{1}$H NMR (CDCl${}_{3}$, 500 MHz), δ [ppm]: 1.17 (d, 3H, *J* = 6.8 Hz, CH(CH${}_{3}$)), 2.40 (dd, 1H, $J_{1}$ = 15.3 Hz, $J_{2}$ = 7.2 Hz, CH_a_H_b_), 2.47 (dd, 1H, $J_{1}$ = 15.3 Hz, $J_{2}$ = 7.2 Hz, CH_a_H_b_), 2.79–2.90 (m, 1H, CH(CH${}_{3}$)), 6.05 (dd, 1H, $J_{1}$ = 15.9 Hz, $J_{2}$ = 7.5 Hz, CH = CH), 6.38 (d, 1H, *J* = 15.9 Hz, CH = CH), 6.93–7.02 (m, 2H, HAr), 7.27–7.32 (m, 2H, HAr), 11.06 (s, 1H, COOH); ${}^{13}$C NMR (125 MHz, CDCl${}_{3}$), δ [ppm]: 20.19 (CH(CH${}_{3}$)), 33.65 (CH(CH${}_{3}$)), 41.36 (CH${}_{2}$), 127.97 (CH = CH), 133.56 (CH = CH), 178.60 (C = O), CAr: 115.37 (d, ${}^{2}J_{F-C}$ = 21.6 Hz), 127.61 (d, ${}^{3}J_{F-C}$ = 7.8 Hz), 133.44 (d, ${}^{4}J_{F-C}$ = 3.3 Hz), 162.11 (d, $J_{F-C}$ = 246.20 Hz); IR (cm${}^{-1}$): 2622, 1698, 1593, 1508, 1461, 1431, 1411, 1378, 1354, 1314, 1261, 1221, 1190, 1160, 1128, 1100, 1079, 1012, 974, 960, 952, 935, 855, 812, 770, 703; HR-MS (ESI-TOF) calculated for C${}_{12}$H${}_{13}$FO${}_{2}$, *m*/*z* [M + Na]${}^{+}$: 231.079723; experimental value: 231.080843.

### 3.4. The Chlorolactonization of Unsaturated Carboxylic Acids **4a**–**c**

The mixture of an unsaturated carboxylic acid (0.005 mol) and *N*-chlorosuccinimide (0.009 mol) was dissolved in 60 mL of THF. Acetic acid was added dropwise and the reaction mixture was stirred at room temperature for 48 h. When the reaction was completed, the mixture was dissolved in diethyl ether, washed with saturated $NaHCO_{3}$ and dried over anhydrous magnesium sulfate. The crude product was purified by column chromatography on silica gel using a mixture of acetone and hexane (1:10) [28]. Spectral data are given below. The chlorolactonization of **4a** gave chlorolactones **5a** and **6a** with a total yield of 51.19% (**5a**:**6a** 81.55:18.45). The chlorolactonization of **4b** gave chlorolactones **5b** and **6b** with a total yield of 82.21% (**5b**:**6b** 84.42:15.58). The chlorolactonization of **4c** gave chlorolactones **5c** and **6c** with a total yield of 61.67% (**5c**:**6c** 81.02:18.98).

*Trans,trans*-5-chloro-tetrahydro-4-methyl-6-(naphthalen-1-yl)pyran-2-one (**5a**): The product was obtained as a colorless solid; mp = 157–158 ${}^{\circ }$C; $R_{f}$ = 0.19 (acetone:hexane 1:7); ${}^{1}$H NMR (CDCl${}_{3}$, 500 MHz), δ [ppm]: 1.29 (d, 3H, *J* = 6.4 Hz, CH(CH${}_{3}$)), 2.47–2.61 (m, 2H, CH(CH${}_{3}$), CH_a_H_b_), 3.07 (dd, 1H, $J_{1}$ = 18.5 Hz, $J_{2}$ = 10.0 Hz, CH_a_H_b_), 4.18 (t, 1H, *J* = 9.7 Hz, CHCl), 5.99 (d, 1H, *J* = 10.0 Hz, CHAr), 7.47–7.61 (m, 4H, HAr), 7.90 (d, 2H, *J* = 8.3 Hz, HAr), 8.09 (d, 1H, *J* = 8.5 Hz, HAr); ${}^{13}$C NMR (125 MHz, CDCl${}_{3}$), δ [ppm]: 19.70 (CH(CH${}_{3}$)), 35.98 (CH(CH${}_{3}$)), 37.15 (CH${}_{2}$), 62.72 (CHCl), 81.97 (CHAr), 169.03 (C = O), CAr: 123.01, 125.00, 125.85, 125.97, 126.62, 129.09, 130.10, 131.18, 131.85, 133.87; IR (cm${}^{-1}$): 1727, 1596, 1512, 1453, 1408, 1375, 1350, 1324, 1286, 1263, 1239, 1216, 1175, 1105, 1085, 1062, 1022, 997, 950, 900, 860, 834, 805, 775, 732, 674, 640; HR-MS (ESI-TOF) calculated for $C_{16}H_{15}ClO_{2}$, *m*/*z* [M + K]${}^{+}$: 313.039760; experimental value: 313.041132.

*Cis,trans*-5-chloro-tetrahydro-4-methyl-6-(naphthalen-1-yl)pyran-2-one (**6a**): The product was obtained as a colorless solid; mp = 98–99.5 ${}^{\circ }$C; $R_{f}$ = 0.28 (acetone:hexane 1:7); ${}^{1}$H NMR (CDCl${}_{3}$, 500 MHz), δ [ppm]: 0.98 (d, 3H, *J* = 6.5 Hz, CH(CH${}_{3}$)), 2.03–2.11 (m, 1H, CH(CH${}_{3}$)), 2.67 (dd, 1H, $J_{1}$ = 18.7 Hz, $J_{2}$ = 11.4 Hz, CH_a_H_b_), 2.75 (dd, 1H, $J_{1}$ = 18.7 Hz, $J_{2}$ = 6.3 Hz, CH_a_H_b_), 4.51 (t, 1H, *J* = 1.9 Hz, CHCl), 6.55 (d, 1H, *J* = 1.9 Hz, CHAr), 7.44–7.59 (m, 4H, HAr), 7.89 (d, 2H, *J* = 8.2 Hz, HAr), 8.07 (d, 1H, *J* = 8.4 Hz, HAr); ${}^{13}$C NMR (125 MHz, CDCl${}_{3}$), δ [ppm]: 17.81 (CH(CH${}_{3}$)), 27.12 (CH(CH${}_{3}$)), 33.06 (CH${}_{2}$), 62.19 (CHCl), 84.33 (CHAr), 169.24 (C = O), CAr: 121.76, 123.56, 125.22, 126.30, 127.35, 129.46, 129.51, 130.12, 132.99, 133.83; IR (cm${}^{-1}$): 1733, 1598, 1507, 1455, 1393, 1342, 1281, 1218, 1169, 1112, 1074, 1038, 997, 945, 909, 886, 863, 802, 777, 737, 703, 615; HR-MS (ESI-TOF) calculated for $C_{16}H_{15}ClO_{2}$, *m*/*z* [M + Na]${}^{+}$: 297.065822; experimental value: 297.067366.

*Trans,trans*-5-chloro-tetrahydro-4-methyl-6-phenylpyran-2-one (**5b**): The product was obtained as a colorless solid; mp = 105–106.5 ${}^{\circ }$C, $R_{f}$ = 0.18 (acetone:hexane 1:7); ${}^{1}$H NMR (CDCl${}_{3}$, 500 MHz), δ [ppm]: 1.25 (d, 3H, *J* = 6.4 Hz, CH(CH${}_{3}$)), 2.35–2.47 (m, 2H, *J* = 17.2 Hz, CH_a_H_b_), 2.92–3.01 (m, 1H, CH_a_H_b_), 3.76 (t, 1H, *J* = 9.8Hz, CHCl), 5.18 (d, 1H, *J* = 10.0 Hz, CHAr), 7.33–7.43 (m, 5H, HAr); ${}^{13}$C NMR (125 MHz, CDCl${}_{3}$), δ [ppm]: 19.62 (CH(CH${}_{3}$)), 35.22 (CH(CH${}_{3}{)}_{2}$), 37.10 (CH${}_{2}$), 63.27 (CHCl), 85.05 (CHAr), 168.95 (C = O), CAr: 127.51, 128.52, 129.29, 136.44; IR (cm${}^{-1}$): 1730, 1496, 1460, 1401, 1376, 1345, 1288, 1240, 1218, 1184, 1107, 1074, 1055, 1031, 1020, 995, 930, 906, 893, 850, 800, 757, 670, 651, 618; HR-MS (ESI-TOF) calculated for $C_{12}H_{13}ClO_{2}$, *m*/*z* [M + Na]${}^{+}$: 247.050173; experimental value: 247.051037.

*Cis,trans*-5-chloro-tetrahydro-4-methyl-6-phenylpyran-2-one (**6b**): The product was obtained as a colorless oil; $n_{D}^{20}$ = 1.5453; $R_{f}$ = 0.27 (acetone:hexane 1:7); ${}^{1}$H NMR (CDCl${}_{3}$, 500 MHz), δ [ppm]: 1.05 (d, 3H, *J* = 6.6 Hz, CH(CH${}_{3}$)), 2.24–2.36 (m, 1H, CH(CH${}_{3}$)), 2.59 (dd, 1H, $J_{1}$ = 18.4 Hz, $J_{2}$ = 10.3 Hz, CH_a_H_b_), 2.64 (dd, 1H, $J_{1}$ = 18.4 Hz, $J_{2}$ = 6.9 Hz, CH_a_H_b_), 4.33 (t, 1H, *J* = 2.5 Hz, CHCl), 5.79 (d, 1H, *J* = 2.5 Hz, CHAr), 7.25–7.28 (m, 2H, HAr), 7.32–7.47 (m, 3H, HAr); ${}^{13}$C NMR (125 MHz, CDCl${}_{3}$), δ [ppm]: 17.65 (CH(CH${}_{3}$)), 27.07 (CH(CH${}_{3}{)}_{2}$), 33.54 (CH${}_{2}$), 63.32 (CHCl), 85.20 (CHAr), 168.97 (C = O), CAr: 125.30, 128.67, 129.00, 137.80; IR (cm${}^{-1}$): 1736, 1949, 1453, 1415, 1370, 1333, 1286, 1243, 1220, 1167, 1112, 1071, 1031, 1004, 949, 909, 881, 860, 830, 802, 757, 710, 699, 606; HR-MS (ESI-TOF) calculated for $C_{12}H_{13}ClO_{2}$, *m*/*z* [M + Na]${}^{+}$: 247.050173; experimental value: 247.050609.

*Trans,trans*-5-chloro-6-(4-fluorophenyl)-tetrahydro-4-methylpyran-2-one (**5c**): The product was obtained as a colorless solid; mp = 80–81 ${}^{\circ }$C; $R_{f}$ = 0.16 (acetone:hexane 1:7); ${}^{1}$H NMR (CDCl${}_{3}$, 500 MHz), δ [ppm]: 1.25 (d, 3H, *J* = 6.3 Hz, CH(CH${}_{3}$)), 2.34–2.49 (m, 2H, CH_a_H_b_, CH(CH${}_{3}$)), 2.94–3.05 (m, 1H, CH_a_H_b_), 3.71 (t, 1H, *J* = 9.8 Hz, CHCl), 5.17 (d, 1H, *J* = 10.0 Hz, CHAr), 7.03–7.13 (m, 2H, HAr), 7.32–7.42 (m, 2H, HAr); ${}^{13}$C NMR (125 MHz, CDCl${}_{3}$), δ [ppm]: 19.62 (CH(CH${}_{3}$)), 35.51 (CH(CH${}_{3}{)}_{2}$), 37.04 (CH${}_{2}$), 63.25 (CHCl), 84.32 (CHAr), 168.71 (C = O), CAr: 115.53 (d, ${}^{2}J_{F-C}$ = 21.9 Hz), 129.35 (d, ${}^{3}J_{F-C}$ = 8.4 Hz), 132.33 (d, ${}^{4}J_{F-C}$ = 3.2 Hz), 163.10 (d, $J_{F-C}$ = 248.4 Hz); IR (cm${}^{-1}$): 1731, 1609, 1514, 1453, 1435, 1427, 1381, 1352, 1334, 1260, 1221, 1192, 1160, 1105, 1083, 1065, 1035, 1010, 960, 919, 883, 844, 825, 810, 786, 719, 687; HR-MS (ESI-TOF) calculated for $C_{12}H_{12}ClFO_{2}$, *m*/*z* [M + Na]${}^{+}$: 265.040751; experimental value: 265.041057.

*Cis,trans*-5-chloro-6-(4-fluorophenyl)-tetrahydro-4-methylpyran-2-one (**6c**): The product was obtained as a colorless oil; $n_{D}^{20}$ = 1.5453, $R_{f}$ = 0.27 (acetone:heksane 1:7); ${}^{1}$H NMR (CDCl${}_{3}$, 500 MHz), δ [ppm]: 1.10 (d, 3H, *J* = 6.6 Hz, CH(CH${}_{3}$)), 2.27–2.37 (m, 1H, CH(CH${}_{3}$)), 2.62 (dd, 1H, $J_{1}$ = 18.4 Hz, $J_{2}$ = 9.7 Hz, CH_a_H_b_), 2.66 (dd, 1H, $J_{1}$ = 18.4 Hz, $J_{2}$ = 7.0 Hz, CH_a_H_b_), 4.30 (t, 1H, *J* = 2.8 Hz, CHCl), 5.75 (d, 1H, *J* = 2.7 Hz, CHAr), 7.09–7.15 (m, 2H, HAr), 7.23–7.33 (m, 2H, HAr); ${}^{13}$C NMR (125 MHz, CDCl${}_{3}$), δ [ppm]: 17.42 (CH(CH${}_{3}$)), 27.41 (CH(CH${}_{3}{)}_{2}$), 33.71 (CH${}_{2}$), 63.02 (CHCl), 84.46 (CHAr), 168.69 (C = O), CAr: 116.02 (d, ${}^{2}J_{F-C}$ = 21.9 Hz), 127.29 (d, ${}^{3}J_{F-C}$ = 8.3 Hz), 133.56 (d, ${}^{4}J_{F-C}$ = 3.1 Hz), 163.65 (d, $J_{F-C}$ = 248.4 Hz); IR (cm${}^{-1}$): 1737, 1605, 1510, 1453, 1414, 1372, 1358, 1340, 1286, 1272, 1218, 1176, 1155, 1110, 1096, 1078, 1060, 1040, 1000, 950, 911, 888, 860, 834, 796, 745, 723, 692, 631; HR-MS (ESI-TOF) calculated for $C_{12}H_{12}ClFO_{2}$, *m*/*z* [M + Na]${}^{+}$: 265.040751; experimental value: 265.041871. 

The bromolactonization of unsaturated carboxylic acids **4a**–**c**: The mixture of an unsaturated carboxylic acid (0.005 mol) and *N*-bromosuccinimide (0.01 mol) was dissolved in 60 mL of THF. Acetic acid was added dropwise and reaction mixture was stirred at room temperature for 48 h. When the reaction was completed, the mixture was dissolved in diethyl ether, washed with saturated NaHCO${}_{3}$ and dried over anhydrous magnesium sulfate. The crude product was purified by column chromatography on silica gel using a mixture of acetone and hexane (1:10) [28]. Spectral data are given below. The bromolactonization of **4a** gave bromolactones **7a** and **8a** with a total yield of 72.23% (**7a**:**8a** 82.30:17.70). The bromolactonization of **4b** gave bromolactones **7b** and **8b** with a total yield of 71.94% (**7b**:**8b** 78.15:21.85). The bromolactonization of **4c** gave bromolactones **7c** and **8c** with a total yield of 77.72% (**7c**:**8c** 76.27:23.73).

*Trans,trans*-5-bromo-tetrahydro-4-methyl-6-(naphthalen-1-yl)pyran-2-one (**7a**): The product was obtained as a colorless solid; mp = 197–198 ${}^{\circ }$C; $R_{f}$ = 0.13 (acetone:hexane 1:7); ${}^{1}$H NMR (CDCl${}_{3}$, 500 MHz), δ [ppm]: 1.33 (d, 3H, *J* = 6.6 Hz, CH(CH${}_{3}$)), 2.55 (dd, 1H, $J_{1}$ = 17.4 Hz, $J_{2}$ = 10.1 Hz, CH_a_H_b_), 2.59–2.72 (m, 1H, CH(CH${}_{3}$)), 3.08 (dd, 1H, $J_{1}$ = 17.4 Hz, $J_{2}$ = 5.7 Hz, CH_a_H_b_), 4.28 (dd, 1H, $J_{1}$ = 10.3 Hz, $J_{2}$ = 9.6 Hz, CHBr), 6.12 (d, 1H, *J* = 10.3 Hz, CHAr), 7.49–7.53 (m, 2H, HAr), 7.53–7.59 (m, 4H, HAr), 7.88–7.93 (m, 2H, HAr), 8.10 (d, 1H, *J* = 8.6 Hz, HAr); ${}^{13}$C NMR (125 MHz, CDCl${}_{3}$), δ [ppm]: 21.13 (CH(CH${}_{3}$)), 36.34 (CH(CH${}_{3}$)), 37.49 (CH${}_{2}$), 54.75 (CHBr), 82.12 (CHAr), 169.22 (C = O), CAr: 123.06, 125.01, 125.89, 126.08, 126.65, 129.14, 130.19, 131.14, 132.24, 133.90; IR (cm${}^{-1}$): 1724, 1596, 1514, 1453, 1413, 1400, 1376, 1345, 1324, 1285, 1236, 1187, 1170, 1105, 1062, 1013, 994, 947, 906, 893, 860, 834, 800, 773, 735, 660, 631; HR-MS (ESI-TOF) calculated for C${}_{16}$H${}_{15}$BrO${}_{2}$, *m*/*z* [M + Na]${}^{+}$: 341.015305; experimental value: 341.016902.

*Cis,trans*-5-bromo-tetrahydro-4-methyl-6-(naphthalen-1-yl)pyran-2-one (**8a**): The product was obtained as a yellow oil, $n_{D}^{20}$ = 1.5140, $R_{f}$ = 0.22 (acetone:hexane 1:7); ${}^{1}$H NMR (CDCl${}_{3}$, 500 MHz), δ [ppm]: 0.96 (d, 3H, *J* = 6.4 Hz, CH(CH${}_{3}$)), 2.04–2.12 (m, 1H, CH(CH${}_{3}$)), 2.66 (dd, 1H, $J_{1}$ = 18.5 Hz, $J_{2}$ = 11.4 Hz, CH_a_H_b_), 2.72 (ddd, 1H, $J_{1}$ = 18.5 Hz, $J_{2}$ = 6.3 Hz, $J_{3}$ = 1.0 Hz, CH_a_H_b_), 4.62 (m, 1H, CHBr), 6.65 (d, 1H, *J* = 1.7 Hz, CHAr), 7.33–7.71 (m, 4H, HAr), 7.83 (d, 1H, *J* = 8.2 Hz, HAr), 7.88 (d, 1H, *J* = 8.2 Hz, HAr), 7.92–7.96 (m, 1H, HAr); ${}^{13}$C NMR (125 MHz, CDCl${}_{3}$), δ [ppm]: 19.26 (CH(CH${}_{3}$)), 27.13 (CH(CH${}_{3}$)), 34.27 (CH${}_{2}$), 56.19 (CHBr), 84.74 (CHAr), 169.10 (C = O), CAr: 121.81, 123.52, 125.22, 126.32, 127.41, 129.15, 129.47, 129.52, 133.60, 133.85; IR (cm${}^{-1}$): 1740, 1596, 1507, 1453, 1390, 1340, 1270, 1236, 1200, 1153, 1107, 1076, 1037, 994, 947, 904, 886, 861, 800, 780, 746, 683, 606; HR-MS (ESI-TOF) calculated for C${}_{16}$H${}_{15}$BrO${}_{2}$, *m*/*z* [M + K]${}^{+}$: 356.989243; experimental value: 356.990538.

*Trans,trans*-5-bromo-tetrahydro-4-methyl-6-phenylpyran-2-one (**7b**): The product was obtained as a colorless solid; mp = 111–112 ${}^{\circ }$C; $R_{f}$ = 0.16 (acetone:hexane 1:7); ${}^{1}$H NMR (CDCl${}_{3}$, 500 MHz), δ [ppm]: 1.28 (d, 3H, *J* = 6.5 Hz, CH(CH${}_{3}$)), 2.44 (dd, 1H, $J_{1}$ = 17.2 Hz, $J_{2}$ = 10.0 Hz, CH_a_H_b_), 2.47–2.59 (m, 1H, CH(CH${}_{3}$)), 2.99 (dd, 1H, $J_{1}$ = 17.2 Hz, $J_{2}$ = 5.6 Hz, CH_a_H_b_), 3.86 (dd, 1H, $J_{1}$ = 10.2 Hz, $J_{2}$ = 9.7 Hz, CHBr), 5.32 (d, 1H, *J* = 10.3 Hz, CHAr), 7.34–7.44 (m, 5H, HAr); ${}^{13}$C NMR (125 MHz, CDCl${}_{3}$), δ [ppm]: 21.04 (CH(CH${}_{3}$)), 35.84 (CH(CH${}_{3}{)}_{2}$), 37.39 (CH${}_{2}$), 55.44 (CHBr), 85.36 (CHAr), 169.19 (C = O); CAr: 127.59, 128.55, 129.36, 136.70; IR (cm${}^{-1}$): 1720, 1497, 1458, 1372, 1345, 1284, 1248, 1184, 1170, 1103, 1061, 1006, 956, 906, 889, 845, 775, 753, 696, 631, 617; HR-MS (ESI-TOF) calculated for C${}_{12}$H${}_{13}$BrO${}_{2}$, *m*/*z* [M + Na]${}^{+}$: 290.999656; experimental value: 291.000717.

*Cis,trans*-5-bromo-tetrahydro-4-methyl-6-phenylpyran-2-one (**8b**): The product was obtained as a yellow oil; $n_{D}^{20}$ = 1.5140, $R_{f}$ = 0.23 (acetone:hexane 1:7); ${}^{1}$H NMR (CDCl${}_{3}$, 500 MHz), δ [ppm]: 1.04 (d, 3H, *J* = 6.5 Hz, CH(CH${}_{3}$)), 2.05–2.14 (m, 1H, CH(CH${}_{3}$)), 2.59 (dd, 1H, $J_{1}$ = 18.4 Hz, $J_{2}$ = 10.8 Hz, CH_a_H_b_), 2.66 (dd, 1H, $J_{1}$ = 18.4 Hz, $J_{2}$ = 6.0 Hz, CH_a_H_b_), 4.46 (t, 1H, *J* = 2.5 Hz, CHBr), 5.89 (d, 1H, *J* = 2.5 Hz, CHAr), 7.27–7.45 (m, 5H, HAr); ${}^{13}$C NMR (125 MHz, CDCl${}_{3}$), δ [ppm]: 19.07 (CH(CH${}_{3}$)), 27.18 (CH(CH${}_{3}{)}_{2}$), 34.69 (CH${}_{2}$), 57.01 (CHBr), 85.53 (CHAr), 168.80 (C = O), CAr: 125.35, 128.67, 129.01, 138.31; IR (cm${}^{-1}$): 1742, 1598, 1451, 1370, 1338, 1281, 1270, 1239, 1162, 1105, 1067, 1035, 1001, 954, 920, 850, 800, 764, 740, 701, 683; HR-MS (ESI-TOF) calculated for C${}_{12}$H${}_{13}$BrO${}_{2}$, *m*/*z* [M + K]${}^{+}$: 306.973594; experimental value: 306.974724.

*Trans,trans*-5-bromo-6-(4-fluorophenyl)-tetrahydro-4-methylpyran-2-one (**7c**): The product was obtained as a colorless solid; mp = 123–124 ${}^{\circ }$C; $R_{f}$ = 0.12 (acetone:hexane 1:7); ${}^{1}$H NMR (CDCl${}_{3}$, 500 MHz), δ [ppm]: 1.28 (d, 3H, *J* = 6.5 Hz, CH(CH${}_{3}$)), 2.44 (dd, 1H, $J_{1}$ = 17.2 Hz, $J_{2}$ = 10.0 Hz, CH_a_H_b_), 2.47–2.61 (m, 1H, CH(CH${}_{3}$)), 2.99 (dd, 1H, $J_{1}$ = 17.2 Hz, $J_{2}$ = 5.6 Hz, CH_a_H_b_), 3.80 (dd, 1H, $J_{1}$ = 10.4 Hz, $J_{2}$ = 9.7 Hz, CHBr), 5.31 (d, 1H, *J* = 10.4 Hz, CHAr), 7.04–7.16 (m, 2H, HAr), 7.32–7.42 (m, 2H, HAr); ${}^{13}$C NMR (125 MHz, CDCl${}_{3}$), δ [ppm]: 21.07 (CH(CH${}_{3}$)), 35.88 (CH(CH${}_{3}{)}_{2}$), 37.33 (CH${}_{2}$), 55.53 (CHBr), 84.64 (CHAr), 168.93 (C = O), CAr: 115.56 (d, ${}^{2}J_{F-C}$ = 21.8 Hz), 129.48 (d, ${}^{3}J_{F-C}$ = 8.5 Hz), 132.77 (d, ${}^{4}J_{F-C}$ = 3.3 Hz), 163.14 (d, $J_{F-C}$ = 248.6 Hz); IR (cm${}^{-1}$): 1729, 1607, 1512, 1454, 1424, 1372, 1331, 1261, 1220, 1190, 1160, 1035, 1004, 850, 918, 877, 840, 823, 790, 773, 719, 660; HR-MS (ESI-TOF) calculated for C${}_{12}$H${}_{12}$BrO${}_{2}$, *m*/*z* [M + Na]${}^{+}$: 308.990234; experimental value: 308.991651.

*Cis,trans*-5-bromo-6-(4-fluorophenyl)-tetrahydro-4-methylpyran-2-one (**8c**): The product was obtained as a colorless oil; $n_{D}^{20}$ = 1.5140, $R_{f}$ = 0.21 (acetone:hexane 1:7); ${}^{1}$H NMR (CDCl${}_{3}$, 500 MHz), δ [ppm]: 1.07 (d, 3H, *J* = 6.5 Hz, CH(CH${}_{3}$)), 2.08–2.12 (m, 1H, CH(CH${}_{3}$)), 2.60 (dd, 1H, $J_{1}$ = 18.4 Hz, $J_{2}$ = 10.3 Hz, CH_a_H_b_), 2.67 (ddd, 1H, $J_{1}$ = 18.4 Hz, $J_{2}$ = 6.1 Hz, $J_{3}$ = 0.9 Hz, CH_a_H_b_), 4.41 (td, 1H, $J_{1}$ = 3.1 Hz, $J_{2}$ = 0.8 Hz, CHBr), 5.84 (dd, 1H, $J_{1}$ = 3.1 Hz, $J_{2}$ = 0.5 Hz CHAr), 7.08–7.13 (m, 2H, HAr), 7.24–7.29 (m, 2H, HAr); ${}^{13}$C NMR (125Hz, CDCl${}_{3}$), δ [ppm]: 18.84 (CH(CH${}_{3}$)), 27.54 (CH(CH${}_{3}{)}_{2}$), 34.84 (CH${}_{2}$), 56.58 (CHBr), 84.80 (CHAr), 168.63 (C = O), CAr: 116.06 (d, ${}^{2}J_{F-C}$ = 21.9 Hz), 127.38 (d, ${}^{3}J_{F-C}$ = 8.3 Hz), 134.02 (d, ${}^{4}J_{F-C}$ = 3.2 Hz), 162.67 (d, $J_{F-C}$ = 248.6 Hz); IR (cm${}^{-1}$): 1740, 1602, 1507, 1453, 1413, 1370, 1359, 1338, 1273, 1227, 1190, 1160, 1115, 1096, 1074, 1062, 1033, 1004, 951, 911, 855, 832, 802, 790, 750, 716, 670; HR-MS (ESI-TOF) calculated for C${}_{12}$H${}_{12}$BrO${}_{2}$, *m*/*z* [M + Na]${}^{+}$: 308.990234; experimental value: 308.991301. 

The iodolactonization of unsaturated carboxylic acids **4a–c**: The mixture of an unsaturated carboxylic acid (0.005 mol), diethyl ether and saturated aqueous $NaHCO_{3}$ was stirred for 30 min and a solution of iodine in potassium iodide was dropwise added. The reaction mixture was refluxed for 24 h. When the reaction was completed, the mixture was dissolved in diethyl ether and washed with saturated $Na_{2}S_{2}O_{3}$ solution. The organic layer was washed with saturated $NaHCO_{3}$ and dried over anhydrous magnesium sulfate. The crude product was purified by column chromatography on silica gel using a mixture of acetone and hexane (1:15) [29]. Spectral data are given below. The iodolactonization of **4a** gave γ-hydroxylactones **12a** and **13a** with a total yield of 84.59% (**12a**:**13a** 10.24:89.76). The iodolactonization of **4b** gave δ-iodolactones **9b**, **10b**, γ-iodoactone **11b** and γ-hydroxylactone **12b**, **13b** with total yield of 75.28% (**9b**:**10b**:**11b**:**12b**:**13b** 41.71:33.19:7.00:13.79:4.65). The iodolactonization of **4c** gave δ-iodolactones **9c**, **10c**, γ-iodoactone **11c** and γ-hydroxylactone **12c**, **13c** with total yield of 68.55% (**9c**:**10c**:**11c**:**12c**:**13c** 30.26:37.42:8.00:7.23:18.08).

*Trans*-dihydro-5-((S)-hydroxy(naphthalen-1-yl)methyl)-4-methylfuran-2(3H)-one (**12a**): The product was obtained as a yellow oil; $n_{D}^{20}$ = 1.427; $R_{f}$ = 0.08 (acetone:hexane 1:7); ${}^{1}$H NMR (CDCl${}_{3}$, 500 MHz), δ [ppm]: 0.56 (d, 3H, *J* = 6.8 Hz, CH${}_{3}$), 2.11 (dd, 1H, $J_{1}$ = 17.5 Hz, $J_{2}$ = 6.6 Hz, CH_a_H_b_), 2.72–2.79 (m, 1H, CH(CH${}_{3}$)), 2.85 (dd, 1H, $J_{1}$ = 17.5 Hz, $J_{2}$ = 9.3 Hz, CH_a_H_b_), 4.53 (dd, 1H, $J_{1}$ = 5.5 Hz, $J_{2}$ = 2.8 Hz, COCH), 5.92 (d, 1H, *J* = 2.3 Hz, CHOH), 7.50–7.59 (m, 3H, HAr), 7.80–7.86 (m, 2H, HAr), 7.90 (d, 1H, *J* = 8.1 Hz, HAr), 7.96 (d, 1H, *J* = 8.1 Hz, HAr); ${}^{13}$C NMR (125 MHz, CDCl${}_{3}$), δ [ppm]: 19.74 (CH${}_{3}$), 28.38 (CH(CH${}_{3}$)), 37.34 (CH${}_{2}$), 69.97 (CHOH), 88.20 (COCH), 175.91 (C = O), CAr: 122.11, 123.65, 125.49, 125.82, 126.64, 128.69, 129.18, 129.88, 133.56, 133.69; IR (cm${}^{-1}$): 3422, 1774, 1593, 1510, 1458, 1417, 1379, 1359, 1327, 1291, 1254, 1209, 1166, 1112, 1092, 1078, 1037, 1008, 975, 936, 920, 881, 854, 823, 802, 782, 741, 694, 660, 628, 615; HR-MS (ESI-TOF) calculated for C${}_{16}$H${}_{16}$O${}_{3}$, *m*/*z* [M + Na]${}^{+}$: 279.099709; experimental value: 279.100786.

*Cis*-dihydro-5-((S)-hydroxy(naphthalen-1-yl)methyl)-4-methylfuran-2(3H)-one (**13a**): The product was obtained as a brown solid; mp = 123–124 ${}^{\circ }$C; $R_{f}$ = 0.06 (acetone:hexane 1:7); ${}^{1}$H NMR (CDCl${}_{3}$, 500 MHz), δ [ppm]: 1.31 (d, 3H, *J* = 7.0 Hz, CH${}_{3}$), 1.57 (s, 1H, OH), 2.57 (dd, 1H, $J_{1}$ = 17.0 Hz, $J_{2}$ = 8.8 Hz, CH_a_H_b_), 2.64 (dd, 1H, $J_{1}$ = 17.0 Hz, $J_{2}$ = 8.6 Hz, CH_a_H_b_), 2.72–2.83 (m, 1H, CH(CH${}_{3}$)), 4.86 (dd, 1H, $J_{1}$ = 7.2 Hz, $J_{2}$ = 3.4 Hz, COCH), 5.72 (t, 1H, *J* = 3.5 Hz, CHOH), 7.47–7.58 (m, 3H, HAr), 7.71 (d, 1H, *J* = 6.9 Hz, HAr), 7.84 (d, 1H, *J* = 8.2 Hz, HAr), 7.90 (d, 1H, *J* = 8.0 Hz, HAr), 8.02 (d, 1H, *J* = 7.9 Hz, HAr); ${}^{13}$C NMR (125 MHz, CDCl${}_{3}$), δ [ppm]: 14.30 (CH${}_{3}$), 33.02 (CH(CH${}_{3}$)), 36.87 (CH${}_{2}$), 68.97 (CHOH), 83.96 (COCH), 177.11 (C = O), CAr: 122.28, 122.95, 125.49, 125.76, 126.58, 129.02, 129.24, 130.36, 133.88, 134.90; IR (cm${}^{-1}$): 3492, 1747, 1593, 1507, 1453, 1415, 1374, 1350, 1327, 1286, 1275, 1257, 1214, 1189, 1173, 1153, 1110, 1096, 1037, 1026, 1000, 942, 918, 852, 834, 787, 773, 755, 730, 655, 644, 622; HR-MS (ESI-TOF) calculated for C${}_{16}$H${}_{16}$O${}_{3}$, *m*/*z* [M + K]${}^{+}$: 295.073647; experimental value: 295.074508.

*Trans,trans*-tetrahydro-5-iodo-4-methyl-6-phenylpyran-2-one (**9b**): The product was obtained as a colorless solid; mp = 107–108 ${}^{\circ }$C; $R_{f}$ = 0.15 (acetone:hexane 1:7); ${}^{1}$H NMR (CDCl${}_{3}$, 500 MHz), δ [ppm]: 1.29 (d, 3H, *J* = 6.6 Hz, CH(CH${}_{3}$)), 2.41 (dd, 1H, $J_{1}$ = 17.4 Hz, $J_{2}$ = 9.8 Hz, CH_a_H_b_), 2.48–2.61 (m, 1H, CH(CH${}_{3}$)), 2.93 (dd, 1H, $J_{1}$ = 17.4 Hz, $J_{2}$ = 5.9 Hz, CH_a_H_b_), 3.97 (dd, 1H, $J_{1}$ = 10.9 Hz, $J_{2}$ = 10.0 Hz, CHI), 5.45 (d, 1H, *J* = 10.9 Hz, CHAr), 7.32–7.44 (m, 5H, HAr); ${}^{13}$C NMR (125 MHz, CDCl${}_{3}$), δ [ppm]: 23.57 (CH(CH${}_{3}$)), 36.00 (CH(CH${}_{3}{)}_{2}$), 37.12 (CH${}_{2}$), 38.88 (CHI), 86.82 (CHAr), 169.49 (C = O), CAr: 127.74, 128.50, 129.43, 137.53; IR (cm${}^{-1}$): 1719, 1499, 1453, 1413, 1372, 1340, 1290, 1245, 1211, 1140, 1100, 1070, 996, 950, 933, 907, 887, 850, 769, 743, 695; HR-MS (ESI-TOF) calculated for C${}_{12}$H${}_{13}$IO${}_{2}$, *m*/*z* [M + Na]${}^{+}$: 338.985797 experimental value: 338.986432.

*Cis,trans*-tetrahydro-5-iodo-4-methyl-6-phenylpyran-2-one (**10b**): The product was obtained as a colorless oil, $n_{D}^{20}$ = 1.5227, $R_{f}$ = 0.19 (acetone:hexane 1:7); ${}^{1}$H NMR (CDCl${}_{3}$, 500 MHz), δ [ppm]: 0.97 (d, 3H, *J* = 6.4 Hz, CH(CH${}_{3}$)), 1.27–1.31 (m, 1H, CH(CH${}_{3}$)), 2.49 (dd, 1H, $J_{1}$ = 18.5 Hz, $J_{2}$ = 10.5 Hz, CH_a_H_b_), 2.67 (ddd, 1H, $J_{1}$ = 18.5 Hz, $J_{2}$ = 5.6 Hz, $J_{3}$ = 1.1 Hz, CH_a_H_b_), 4.60 (td, 1H, $J_{1}$ = 3.1 Hz, $J_{2}$ = 1.1 Hz, CHI), 5.91 (d, 1H, *J* = 3.1 Hz, CHAr), 7.25–7.43 (m, 5H, HAr); ${}^{13}$C NMR (125 MHz, CDCl${}_{3}$), δ [ppm]: 21.03 (CH(CH${}_{3}$)), 27.34 (CH(CH${}_{3}{)}_{2}$), 36.36 (CH${}_{2}$), 38.08 (CHBr), 86.32 (CHAr), 168.32 (C = O), CAr: 125.06, 128.18, 128.51, 138.42; IR (cm${}^{-1}$): 1735, 1495, 1451, 1407, 1380, 1368, 1354, 1334, 1322, 1285, 1265, 1233, 1190, 1132, 1103, 1061, 1030, 998, 950, 903, 883, 852, 796, 763, 735, 695, 664; HR-MS (ESI-TOF) calculated for C${}_{12}$H${}_{13}$IO${}_{2}$, *m*/*z* [M + K]${}^{+}$: 354.959735 experimental value: 354.958974.

*Cis*-dihydro-5-(iodo(phenyl)methyl)-4-methylfuran-2(3H)-one (**11b**): The product was obtained as a colorless solid, mp = 86–87 ${}^{\circ }$C; $R_{f}$ = 0.15 (acetone:hexane 1:7); ${}^{1}$H NMR (CDCl${}_{3}$, 500 MHz), δ [ppm]: 1.17 (d, 3H, *J* = 7.0 Hz, CH${}_{3}$), 2.32 (dd, 1H, $J_{1}$ = 17.0 Hz, $J_{2}$ = 0.5 Hz, CH_a_H_b_), 2.87 (dd, 1H, $J_{1}$ = 17.0 Hz, $J_{2}$ = 7.4 Hz, CH_a_H_b_), 2.96–3.03 (m, 1H, CH(CH${}_{3}$)), 4.96 (d, 1H, *J* = 11.2 Hz, CHI), 5.11 (dd, 1H, $J_{1}$ = 11.2 Hz, $J_{2}$ = 4.5 Hz, COCH), 7.34–7.39 (m, 3H, HAr), 7.39–7.44 (m, 2H, HAr); ${}^{13}$C NMR (125 MHz, CDCl${}_{3}$), δ [ppm]: 12.82 (CH${}_{3}$), 28.38 (CHI), 34.00 (CH(CH${}_{3}$)), 38.88 (CH${}_{2}$), 84.45 (COCH), 176.00 (C = O), CAr: 127.79, 128.61, 128.85, 140.09; IR (cm${}^{-1}$): 1765, 1500, 1452, 1414, 1372, 1341, 1290, 1247, 1212, 1141, 1100, 1071, 997, 950, 932, 908, 886, 851, 769, 742, 695; HR-MS (ESI-TOF) calculated for C${}_{12}$H${}_{13}$IO${}_{2}$, *m*/*z* [M + Na]${}^{+}$: 338.985797 experimental value: 338.986737.

*Trans*-dihydro-5-(hydroxy(phenyl)methyl)-4-methylfuran-2(3H)-one (**12b**): The product was obtained as a colorless solid, mp = 86–87 ${}^{\circ }$C; $R_{f}$ = 0.11 (acetone:hexane 1:7); ${}^{1}$H NMR (CDCl${}_{3}$, 500 MHz), δ [ppm]: 0.81 (d, 3H, *J* = 6.9 Hz, CH${}_{3}$), 2.10 (dd, 1H, $J_{1}$ = 17.6 Hz, $J_{2}$ = 6.7 Hz, CH_a_H_b_), 2.57–2.76 (m, 1H, CH(CH${}_{3}$)), 2.73 (dd, 1H, $J_{1}$ = 17.6 Hz, $J_{2}$ = 9.2 Hz, CH_a_H_b_), 4.30 (dd, 1H, $J_{1}$ = 5.5 Hz, $J_{2}$ = 3.3 Hz, COCH), 5.09 (t, 1H, *J* = 3.6 Hz, CHOH), 7.29–7.34 (m, 3H, HAr), 7.36–7.42 (m, 2H, HAr); ${}^{13}$C NMR (125 MHz, CDCl${}_{3}$), δ [ppm]: 19.77 (CH${}_{3}$), 28.80 (CH(CH${}_{3}$)), 37.06 (CH${}_{2}$), 72.29 (CHOH), 89.74 (COCH), 176.99 (C = O), CAr: 126.00, 128.12, 128.63, 138.34; IR (cm${}^{-1}$): 3442, 1767, 1600, 1496, 1451, 1415, 1379, 1356, 1324, 1275, 1225, 1214, 1202, 1162, 1103, 1085, 1060, 1008, 983, 936, 915, 879, 859, 818, 762, 707, 694, 676, 617; HR-MS (ESI-TOF) calculated for C${}_{12}$H${}_{14}$O${}_{3}$, *m*/*z* [M + Na]${}^{+}$: 229.084059 experimental value: 229.084576.

*Cis*-dihydro-5-(hydroxy(phenyl)methyl)-4-methylfuran-2(3H)-one (**13b**): The product was obtained as a colorless solid, mp = 89–91 ${}^{\circ }$C; $R_{f}$ = 0.11 (acetone:hexane 1:7); ${}^{1}$H NMR (CDCl${}_{3}$, 500 MHz), δ [ppm]: 1.32 (d, 3H, *J* = 6.9 Hz, CH${}_{3}$), 1.58 (s, 1H, OH), 2.20 (dd, 1H, $J_{1}$ = 17.2 Hz, $J_{2}$ = 4.8 Hz, CH_a_H_b_), 2.77 (dd, 1H, $J_{1}$ = 17.3 Hz, $J_{2}$ = 8.4 Hz, CH_a_H_b_), 2.80–2.89 (m, 1H, CH(CH${}_{3}$)), 4.85 (dd, 1H, $J_{1}$ = 7.2 Hz, $J_{2}$ = 4.0 Hz, COCH), 5.11 (d, 1H, *J* = 3.9 Hz, CHOH), 7.27–7.35 (m, 2H, HAr), 7.37–7.48 (m, 2H, HAr); ${}^{13}$C NMR (125 MHz, CDCl${}_{3}$), δ [ppm]: 19.20 (CH${}_{3}$), 32.85 (CH(CH${}_{3}$)), 35.03 (CH${}_{2}$), 69.70 (CHOH), 84.59 (COCH), 175.49 (C = O), CAr: 125.67, 128.29, 128.78, 138.37; IR (cm${}^{-1}$): 3419, 1751, 1587, 1497, 1434, 1403, 1387, 1361, 1301, 1255, 1243, 1202, 1167, 1075, 1067, 1054, 1011, 983, 933, 887, 828, 785, 752, 732, 697; HR-MS (ESI-TOF) calculated for C${}_{12}$H${}_{14}$O${}_{3}$, *m*/*z* [M + Na]${}^{+}$: 229.084059 experimental value: 229.084781.

*Trans,trans*-6-(4-fluorophenyl)-tetrahydro-5-iodo-4-methylpyran-2-one (**9c**): The product was obtained as a colorless solid, mp = 136–138 ${}^{\circ }$C, $R_{f}$ = 0.16 (acetone:hexane 1:7); ${}^{1}$H NMR (CDCl${}_{3}$, 500 MHz), δ [ppm]: 1.28 (d, 3H, *J* = 6.5 Hz, CH(CH${}_{3}$)), 2.36 (dd, 1H, $J_{1}$ = 17.2 Hz, $J_{2}$ = 10.0 Hz, CH_a_H_b_), 2.51–2.57 (m, 1H, CH(CH${}_{3}$)), 2.92 (dd, 1H, $J_{1}$ = 17.5 Hz, $J_{2}$ = 6.0 Hz, CH_a_H_b_), 3.90 (dd, 1H, $J_{1}$ = 10.8 Hz, $J_{2}$ = 10.1 Hz, CHI), 5.42 (d, 1H, *J* = 10.8 Hz, CHAr), 7.05–7.09 (m, 2H, HAr), 7.32–7.36 (m, 2H, HAr); ${}^{13}$C NMR (125 MHz, CDCl${}_{3}$), δ [ppm]: 23.6 (CH(CH${}_{3}$)), 27.03 (CH(CH${}_{3}{)}_{2}$), 31.66 (CH${}_{2}$), 37.69 (CHI), 86.43 (CHAr), 169.68 (C = O), CAr: 115.53 (d, ${}^{2}J_{F-C}$ = 21.7 Hz), 129.58 (d, ${}^{3}J_{F-C}$ = 8.4 Hz), 133.78 (d, ${}^{4}J_{F-C}$ = 3.3 Hz), 163.09 (d, $J_{F-C}$ = 248.60 Hz); IR (cm${}^{-1}$): 1720, 1601, 1515, 1459, 1421, 1411, 1366, 1334, 1300, 1285, 1223, 1207, 1176, 1162, 1110, 1090, 1071, 1053, 1004, 952, 909, 896, 858, 844, 822, 778, 775, 717; HR-MS (ESI-TOF) calculated for C${}_{12}$H${}_{12}$FIO${}_{2}$, *m*/*z* [M + Na]${}^{+}$: 356.976375 experimental value: 356.977702.

*Cis*,trans-6-(4-fluorophenyl)-tetrahydro-5-iodo-4-methylpyran-2-one (**10c**): The product was obtained as a yellow oil; $n_{D}^{20}$ = 1.5227, $R_{f}$ = 0.24 (acetone:hexane 1:7); ${}^{1}$H NMR (CDCl${}_{3}$, 500 MHz), δ [ppm]: 1.04 (d, 3H, *J* = 6.5 Hz, CH(CH${}_{3}$)), 1.39–1.48 (m, 1H, CH(CH${}_{3}$)), 2.52 (dd, 1H, $J_{1}$ = 18.4 Hz, $J_{2}$ = 9.8 Hz, CH_a_H_b_), 2.71 (ddd, 1H, $J_{1}$ = 18.4 Hz, $J_{2}$ = 5.5 Hz, $J_{3}$ = 1.0 Hz, CH_a_H_b_), 4.55 (td, 1H, $J_{1}$ = 3.9 Hz, $J_{2}$ = 0.9 Hz, CHI), 5.86 (d, 1H, *J* = 3.9 Hz, CHAr), 7.04–7.13 (m, 2H, HAr), 7.22–7.29 (m, 2H, HAr); ${}^{13}$C NMR (125 MHz, CDCl${}_{3}$), δ [ppm]: 21.13 (CH(CH${}_{3}$)), 28.37 (CH(CH${}_{3}{)}_{2}$), 30.90 (CH${}_{2}$), 36.88 (CHI), 85.92 (CHAr), 168.53 (C = O), CAr: 115.96 (d, ${}^{2}J_{F-C}$ = 21.8 Hz), 127.56 (d, ${}^{3}J_{F-C}$ = 8.3 Hz), 134.62 (d, ${}^{4}J_{F-C}$ = 3.0 Hz), 162.62 (d, $J_{F-C}$ = 248.8 Hz); IR (cm${}^{-1}$): 1735, 1605, 1508, 1453, 1411, 1384, 1366, 1354, 1340, 1328, 1283, 1263, 1225, 1184, 1157, 1133, 1095, 1073, 1056, 998, 946, 907, 890, 851, 829, 802, 782, 750, 714; HR-MS (ESI-TOF) calculated for C${}_{12}$H${}_{12}$FIO${}_{2}$, *m*/*z* [M + Na]${}^{+}$: 356.976375 experimental value: 356.977461.

*Cis*-5-((4-fluorophenyl)iodomethyl)-dihydro-4-methylfuran-2(3H)-one (**11c**): The product was obtained as a colorless solid, mp = 118–119 ${}^{\circ }$C; $R_{f}$ = 0.16 (acetone:hexane 1:7); ${}^{1}$H NMR (CDCl${}_{3}$, 500 MHz), δ [ppm]: 1.16 (d, 3H, *J* = 7.1 Hz, CH${}_{3}$), 2.33 (dd, 1H, $J_{1}$ = 17.1 Hz, $J_{2}$ = 0.5 Hz, CH_a_H_b_), 2.87 (dd, 1H, $J_{1}$ = 17.1 Hz, $J_{2}$ = 7.3 Hz, CH_a_H_b_), 2.95–3.00 (m, 1H, CH(CH${}_{3}$)), 4.94 (d, 1H, $J_{1}$ = 11.2 Hz, CHI), 5.04 (dd, 1H, $J_{1}$ = 11.2 Hz, $J_{2}$ = 4.5 Hz, COCH), 6.97–7.10 (m, 2H, HAr), 7.32–7.43 (m, 2H, HAr); ${}^{13}$C NMR (125 MHz, CDCl${}_{3}$), δ [ppm]: 12.85 (CH${}_{3}$), 28.45 (CHI), 34.05 (CH(CH${}_{3}$)), 38.91 (CH${}_{2}$), 84.30 (COCH), 176.11 (C = O), CAr: 116.24 (d, ${}^{2}J_{F-C}$ = 22.1 Hz), 127.64 (d, ${}^{3}J_{F-C}$ = 8.0 Hz), 133.62 (d, ${}^{4}J_{F-C}$ = 3.0 Hz), 162.38 (d, $J_{F-C}$ = 246.3 Hz). IR (cm${}^{-1}$): 1760, 1600, 1512, 1460, 1420, 1412, 1361, 1335, 1301, 1284, 1226, 1203, 1174, 1161, 1108, 1091, 1070, 1052, 1001, 950, 906, 894, 857, 842, 821, 779, 776, 714; HR-MS (ESI-TOF) calculated for C${}_{12}$H${}_{12}$FIO${}_{2}$, *m*/*z* [M + Na]${}^{+}$: 356.976375 experimental value: 356.976932.

*Trans*-5-((4-fluorophenyl)(hydroxy)methyl)-dihydro-4-methylfuran-2(3H)-one (**12c**): The product was obtained as a colorless solid, mp = 118–119 ${}^{\circ }$C, $R_{f}$ = 0.10 (acetone:hexane 1:7); ${}^{1}$H NMR (CDCl${}_{3}$, 500 MHz), δ [ppm]: 0.83 (d, 3H, *J* = 6.9 Hz, CH${}_{3}$), 2.11 (dd, 1H, $J_{1}$ = 17.7 Hz, $J_{2}$ = 6.9 Hz, CH_a_H_b_), 2.53–2.64 (m, 1H, CH(CH${}_{3}$)), 2.74 (dd, 1H, $J_{1}$ = 17.7 Hz, $J_{2}$ = 9.2 Hz, CH_a_H_b_), 4.26 (dd, 1H, $J_{1}$ = 5.3 Hz, $J_{2}$ = 3.4 Hz, COCH), 5.07 (d, 1H, *J* = 3.2 Hz, CHOH), 6.98–7.12 (m, 2H, HAr), 7.33–7.43 (m, 2H, HAr); ${}^{13}$C NMR (125 MHz, CDCl${}_{3}$), δ [ppm]: 19.74 (CH${}_{3}$), 28.84 (CH(CH${}_{3}$)), 37.03 (CH${}_{2}$), 72.70 (CHOH), 89.65 (COCH), 176.93 (C = O), CAr: 115.57 (d, ${}^{2}J_{F-C}$ = 21.5 Hz), 127.72 (d, ${}^{3}J_{F-C}$ = 8.1 Hz), 134.17 (d, ${}^{4}J_{F-C}$ = 3.1 Hz), 163.46 (d, $J_{F-C}$ = 246.6 Hz); IR (cm${}^{-1}$): 3431, 1772, 1593, 1509, 1456, 1412, 1378, 1361, 1329, 1287, 1257, 1215, 1169, 1114, 1093, 1075, 1041, 1008, 975, 936, 920, 881, 854, 826, 802, 782, 749, 715; HR-MS (ESI-TOF) calculated for C${}_{12}$H${}_{13}$FO${}_{3}$, *m*/*z* [M + Na]${}^{+}$: 247.074638 experimental value: 247.075208.

*Cis*-5-((4-fluorophenyl)(hydroxy)methyl)-dihydro-4-methylfuran-2(3H)-one (**13c**): The product was obtained as a colorless solid, mp = 118–119 ${}^{\circ }$C; $R_{f}$ = 0.08 (acetone:hexane 1:7); ${}^{1}$H NMR (CDCl${}_{3}$, 500 MHz), δ [ppm]: 1.34 (d, 3H, *J* = 6.8 Hz, CH${}_{3}$), 1.59 (s, 1H, OH), 2.24 (dd, 1H, $J_{1}$ = 17.3 Hz, $J_{2}$ = 4.8 Hz, CH_a_H_b_), 2.78 (dd, 1H, $J_{1}$ = 17.3 Hz, $J_{2}$ = 8.5 Hz, CH_a_H_b_), 2.81–2.89 (m, 1H, CH(CH${}_{3}$)), 4.81 (dd, 1H, $J_{1}$ = 7.3 Hz, $J_{2}$ = 4.1 Hz, COCH), 5.09 (d, 1H, *J* = 4.1 Hz, CHOH), 7.14–7.24 (m, 2H, HAr), 7.29–7.38 (m, 2H, HAr); ${}^{13}$C NMR (125 MHz, CDCl${}_{3}$), δ [ppm]: 19.22 (CH${}_{3}$), 32.86 (CH(CH${}_{3}$)), 35.05 (CH${}_{2}$), 69.72 (CHOH), 84.57 (COCH), 175.47 (C = O), CAr: 116.26 (d, ${}^{2}J_{F-C}$ = 22.1 Hz), 127.69 (d, ${}^{3}J_{F-C}$ = 8.0 Hz), 133.63 (d, ${}^{4}J_{F-C}$ = 3.0 Hz), 162.34 (d, $J_{F-C}$ = 246.4 Hz); IR (cm${}^{-1}$): 3439, 1751, 1592, 1507, 1459, 1410, 1380, 1365, 1327, 1285, 1259, 1216, 1169, 1115, 1094, 1077, 1044, 1010, 973, 937, 920, 882, 853, 827, 801, 782, 748, 716; HR-MS. HR-MS (ESI-TOF) calculated for C${}_{12}$H${}_{13}$FO${}_{3}$, *m*/*z* [M + Na]${}^{+}$: 247.074638 experimental value: 247.075284.

### 3.5. Cytotoxic Activity

Cell cultures: The L929 mouse fibroblasts and human gastric adenocarcinoma cell line AGS were used for in vitro cytotoxicity assays. The cells were maintained under standard conditions (37 ${}^{\circ }$C, 5% CO${}_{2}$) in 25 $cm^{2}$ tissue culture flasks in RPMI-1640 medium supplemented with 10% fetal bovine serum (FBS) and antibiotics: 100 U/mL penicillin and 100 μg/mL streptomycin. The cell suspension containing 10${}^{8}$ cells in 1 mL, was obtained by treatment of confluent monolayers with 0.25% trypsin solution, washed and subcultured. Cell cultures were supplemented with medium to maintain them in log phase growth. The viability of the cells was assessed by exclusion of trypan blue dye.

Measurements of cellular metabolic activity and global growth inhibition: L929 cells were seeded into 96-well plates (2 × 10${}^{5}$ cells/well) for 24 h, at 37 ${}^{\circ }$C, 5% $CO_{2}$. Tested compounds **5a**–**c**, **6a**–**c**, **7a**–**c**, **8a**–**c**, **9b,c**, **10b,c**, **12a** and **13a** were diluted serially in RPMI-1640 medium in concentrations of 50, 20, 10, 5, 0.5 and 0.1 μg/mL, added to the cells (100 μL/well), and incubated under standard conditions for 24 h [43]. The potential harmful effect of lactones on L929 and AGS cells was assessed by the ability of the cells to reduce 3-(4,5-dimethylthiazol-2-yl)-2,5-diphenyltetrazolium bromide (MTT) to formazan. The solution of MTT (5 mg/mL in PBS), was added to each well and the plates were incubated for 4h at 37 ${}^{\circ }$C. Formazan crystals were dissolved with acidic isopropanol (0.1 M HCl in absolute isopropanol) and the plates were read in a plate reader (Victor 2) at 570 nm. The percentage of MTT reduction was calculated by comparing the absorbance of treated cells to that of untreated control cells. Results are expressed as the mean percentage of viable cells ± standard deviation (SD) of four independent experiments. Statistical significance was accepted at a *p*-value < 0.05.

### 3.6. Bactericidal Activity

The bacterial cultures: *Escherichia coli* ATCC 8739 and *Staphylococcus aureus* ATCC 65389 were grown on Luria–Bertani Agar for 18 h at 37 ${}^{\circ }$C. The strain suspension was prepared in 0.85% saline, with an optical density equivalent to a 0.5 McFarland standard. Next, the following mixtures were prepared: 180 μL of sterile enriched broth (BTL), 10 μL of the bacterial suspension and 10 μL of the tested compound (range 0.1–50 μg/mL) solubilized in DMSO. As a negative control, only DMSO was added. As a sterility control, 180 μL of BTL and 20 μL saline were used. The microtiter plate was incubated at 37 ${}^{\circ }$C for 18 h. The colony forming unit (CFU) method was used to evaluate the microbial population. The experiment was repeated three times. Gentamicin was used as the reference antibiotic [23].

## 4. Conclusions

A four-step synthesis gave sixteen new γ-halo-δ-lactones, two γ-iodo-δ-lactones and unexpectedly six δ-hydroxy-γ-lactones. The stereoselectivity of the iodolactonization reaction is significant. The yield of *trans,trans* chloro- and bromo-δ-lactone was in the range of 76–82%. The iodolactonization of unsaturated carboxylic acids gave *cis* δ-hydroxy-γ-lactones with a yield of 90%. All obtained lactones were tested for antimicrobial activity against *Escherichia coli* strains ATCC 8739 and *Staphylococcus aureus* ATCC 65389. The δ-iodolactone **10c** and δ-hydroxy-γ-lactones **12a** and **13a** showed high bactericidal activity against *E. coli.* The highest bactericidal activity against *S. aureus* was observed for lactone **5a**, which reduced the number of CFU by 87%. Significant bactericidal activity was observed for *cis,trans*-γ-chloro-δ-lactone **6c** (83%) and *cis,trans*-γ-bromo-δ-lactone **8a** (83%). Synthesized lactones were also tested for cytotoxic activity against two cell lines, mouse fibroblasts L929 and gastric cancer cells AGS. The strongest cell growth inhibition against L929 fibroblasts was observed for *cis,trans*-γ-iodo-δ-lactones **10b,c** and *cis,trans*-γ-chloro-δ-lactone **6b**. The AGS cells were highly sensitive to *trans,trans*-γ-chloro-δ-lactone **5c**, and *trans,trans*-γ-iodo-δ-lactones **9b,c**. Their cytotoxic activity against gastric cancer AGS cells was much higher than doxorubicin, thus these lactones could be promising candidates for anticancer formulations.

## Figures and Tables

**Table 1:** The yields and products distribution from halolactonization of unsaturated carboxylic acids **4a**–**c** (according to GC).

	γ-Chloro-δ-lactones			γ-Bromo-δ-lactones		
	yield [%]	*trans,trans*	*cis,trans*	yield [%]	*trans,trans*	*cis,trans*
$-C_{10}H_{7}$	51	**5a** 82%	**6a** 18%	72	**7a** 82%	**8a** 18%
$-C_{6}H_{5}$	82	**5b** 84%	**6b** 16%	72	**7b** 78%	**8b** 22%
$-C_{6}H_{4}F$	62	**5c** 81%	**6c** 19%	78	**7c** 76%	**8c** 24%

**Table 2:** The yields and products distribution from iodolactonization of unsaturated carboxylic acids **4a**–**c** (according to GC).

	*cis*-δ-Iodo-δ-lactones			γ-Iodo-δ-lactones		δ-Hydroxy-γ-lactones	
	yield [%]	*trans*	*cis*	*trans,trans*	*cis,trans*	*trans*	*cis*
$-C_{10}H_{7}$				-	-	**12a** 10%	**13a** 90%
$-C_{6}H_{5}$	75	-	**11b** 10%	**9b** 42%	**10b** 33%	**12b** 4%	**13b** 11%
$-C_{6}H_{4}F$	69	-	**11c** 8%	**9c** 37%	**10c** 30%	**12c** 7%	**13c** 18%

**Table 3:** The bactericidal activity of the investigated lactones against *Escherichia coli* ATCC 8739 and *Staphylococcus aureus* ATCC 65389.

Compound	*E. coli* ATCC 8739	*S. aureus* ATCC 65389
	Number of CFU/mL (× $10^{7}$)	
**5a**	6.41	2.74
**5b**	4.06	7.63
**5c**	9.46	14.83
**6a**	7.58	5.20
**6b**	6.32	3.58
**6c**	2.06	7.39
**7a**	5.21	4.63
**7b**	8.63	10.96
**7c**	6.52	6.42
**8a**	7.14	9.29
**8b**	5.83	9.04
**8c**	8.87	3.60
**9b**	9.02	21.25
**9c**	2.43	9.76
**10b**	7.65	5.92
**10c**	2.87	6.30
**12a**	9.43	21.15
**13a**	6.02	9.12
Control	9.47	21.36
Control with DMSO	9.30	20.85

**Table 4:** The cytotoxic activity of tested lactones against AGS cell lines.

Lactone	Concentration [μg/mL]						5% DMSO	${\mathrm{IC}}_{50}$ [μM]
	50	20	10	5	0.5	0.1		
**5a**	15 ± 0.01	18 ± 0.08	27 ± 0.10	43 ± 0.09	48 ± 0.05	54 ± 0.07	85 ± 0.1	0.0019
**5b**	8 ± 0.09	11 ± 0.07	14 ± 0.12	16 ± 0.06	20 ± 0.09	35 ± 0.06		0.0023
**5c**	10 ± 0.12	21 ± 0.08	34 ± 0.09	44 ± 0.10	47 ± 0.12	54 ± 0.12		0.0006
**6a**	8 ± 0.05	8 ± 0.09	19 ± 0.01	23 ± 0.09	28 ± 0.07	35 ± 0.06		0.0019
**6b**	8 ± 0.06	14 ± 0.07	21 ± 0.02	28 ± 0.03	35 ± 0.02	42 ± 0.02		0.0044
**6c**	6 ± 0.08	14 ± 0.07	16 ± 0.10	28 ± 0.06	37 ± 0.05	49 ± 0.10		0.0029
**7a**	8 ± 0.03	20 ± 0.06	21 ± 0.03	25 ± 0.10	26 ± 0.05	39 ± 0.02		0.0021
**7b**	10 ± 0.06	14 ± 0.03	20 ± 0.06	23 ± 0.02	25 ± 0.10	40 ± 0.10		0.0036
**7c**	8 ± 0.07	18 ± 0.10	25 ± 0.12	42 ± 0.02	49 ± 0.06	63 ± 0.03		0.0035
**8a**	9 ± 0.09	17 ± 0.07	25 ± 0.05	39 ± 0.09	47 ± 0.06	61 ± 0.10		0.0016
**8b**	11 ± 0.05	17 ± 0.10	28 ± 0.09	31 ± 0.02	35 ± 0.10	44 ± 0.15		0.0019
**8c**	10 ± 0.09	11 ± 0.03	18 ± 0.07	43 ± 0.10	58 ± 0.10	69 ± 0.15		0.0025
**9b**	8 ± 0.09	15 ± 0.06	26 ± 0.03	43 ± 0.12	52 ± 0.15	54 ± 0.15		0.0014
**9c**	15 ± 0.03	18 ± 0.06	23 ± 0.02	35 ± 0.10	43 ± 0.15	51 ± 0.10		0.0014
**10b**	10 ± 0.06	14 ± 0.08	16 ± 0.09	37 ± 0.03	43 ± 0.02	58 ± 0.07		0.0018
**10c**	8 ± 0.03	10 ± 0.07	13 ± 0.03	20 ± 0.02	23 ± 0.15	38 ± 0.06		0.0017
**12a**	9 ± 0.10	13 ± 0.08	18 ± 0.05	22 ± 0.09	26 ± 0.05	39 ± 0.09		0.0043
**13a**	8 ± 0.08	10 ± 0.10	18 ± 0.12	29 ± 0.05	39 ± 0.09	44 ± 0.12		0.0037

The cytotoxicity was assessed by (3-(4,5-dimethylthiazol-2-yl)-2,5-diphenyltetrazolium bromide) (MTT) reduction assay. The cell viability was calculated for four experiments including three repeats for each compound. Complete RPMI-1640 medium was used as a positive control (Control +) of cell viability (100% viable cells) and 0.03% H${}_{2}$O${}_{2}$ as a negative control (Control −) of cell viability (100% dead inactive cells). All values were expressed as the mean percentage of viable cells ± SD. The differences between positive control and treated with tested compounds were evaluated by non-parametric Mann–Whitney U test. Statistical significance: *p* < 0.05. Dimethyl sulfoxide (DMSO) was used as a solvent; $IC_{50}$, half maximal inhibitory concentration.

**Table 5:** The cytotoxic activity of tested lactones against L929 cell lines.

Lactone	Concentration [μg/mL]						5% DMSO	${\mathrm{IC}}_{50}$ [μM]
	50	20	10	5	0.5	0.1		
**5a**	1 ± 0.01	5 ± 0.06	8 ± 0.07	10 ± 0.03	15 ± 0.02	17 ± 0.09	79 ± 0.07	0.0058
**5b**	1 ± 0.03	4 ± 0.02	7 ± 0.06	12 ± 0.05	18 ± 0.15	20 ± 0.08		0.0071
**5c**	2 ± 0.12	4 ± 0.06	10 ± 0.03	14 ± 0.09	21 ± 0.15	26 ± 0.03		0.0078
**6a**	2 ± 0.09	6 ± 0.03	7 ± 0.07	9 ± 0.15	11 ± 0.08	15 ± 0.03		0.0083
**6b**	2 ± 0.05	4 ± 0.15	16 ± 0.03	25 ± 0.02	27 ± 0.12	32 ± 0.07		0.0041
**6c**	2 ± 0.02	4 ± 0.08	9 ± 0.05	18 ± 0.12	22 ± 0.05	24 ± 0.03		0.0047
**7a**	1 ± 0.12	2 ± 0.03	6 ± 0.07	12 ± 0.02	18 ± 0.12	25 ± 0.02		0.0043
**7b**	2 ± 0.09	5 ± 0.15	10 ± 0.05	13 ± 0.12	18 ± 0.08	25 ± 0.03		0.0048
**7c**	1 ± 0.03	5 ± 0.05	11 ± 0.08	17 ± 0.02	21 ± 0.08	27 ± 0.08		0.0066
**8a**	1 ± 0.15	4 ± 0.02	7 ± 0.06	14 ± 0.03	19 ± 0.03	23 ± 0.03		0.0044
**8b**	1 ± 0.02	4 ± 0.08	8 ± 0.05	15 ± 0.03	17 ± 0.08	24 ± 0.03		0.0055
**8c**	1 ± 0.05	5 ± 0.02	11 ± 0.03	15 ± 0.03	20 ± 0.08	25 ± 0.03		0.0044
**9b**	1 ± 0.12	4 ± 0.15	11 ± 0.06	13 ± 0.07	18 ± 0.03	23 ± 0.03		0.0044
**9c**	1 ± 0.05	3 ± 0.09	6 ± 0.03	9 ± 0.06	12 ± 0.08	15 ± 0.03		0.0068
**10b**	1 ± 0.02	5 ± 0.06	7 ± 0.15	15 ± 0.08	20 ± 0.03	25 ± 0.08		0.0040
**10c**	2 ± 0.05	2 ± 0.15	6 ± 0.09	12 ± 0.07	18 ± 0.03	22 ± 0.05		0.0041
**12a**	2 ± 0.06	3 ± 0.03	8 ± 0.06	10 ± 0.02	15 ± 0.05	19 ± 0.15		0.0062
**13a**	1 ± 0.07	5 ± 0.12	6 ± 0.06	9 ± 0.03	12 ± 0.02	16 ± 0.09		0.0081

The cytotoxicity was assessed by (3-(4,5-dimethylthiazol-2-yl)-2,5-diphenyltetrazolium bromide) (MTT) reduction assay. The cell viability was calculated for four experiments including three repeats for each compound. Complete RPMI-1640 medium was used as a positive control (Control +) of cell viability (100% viable cells) and 0.03% H${}_{2}$O${}_{2}$ as a negative control (Control −) of cell viability (100% dead inactive cells). All values were expressed as the mean percentage of viable cells ± SD. The differences between positive control and treated with tested compounds were evaluated by non-parametric Mann–Whitney U test. Statistical significance: *p* < 0.05. Dimethyl sulfoxide (DMSO) was used as a solvent; $IC_{50}$, half maximal inhibitory concentration.

**Table 6:** Experimental details of the crystallographic analysis for lactones **5a**, **7a**, **13a**, **12b** and **12c**.

	5a	7a	13a	12b	12c
Empirical formula	C${}_{16}$H${}_{15}$O${}_{2}$Cl	C${}_{16}$H${}_{15}$O${}_{2}$Br	C${}_{16}$H${}_{16}$O${}_{3}$	C${}_{12}$H${}_{14}$O${}_{3}$	C${}_{12}$H${}_{13}$FO${}_{3}$
Formula weight	274.73	319.19	256.29	206.24	224.22
Temperature (K)	293.15	293.15	100	100	293.15
Wavelength (Å)	0.71073	0.71073	1.54184	1.54184	0.71073
Crystal system, space group	monoclinic, $P2_{1/c}$	monoclinic, $P2_{1/c}$	monoclinic, $P2_{1/c}$	orthorhombic P b c a ($P2_{1}/b2_{1}/c2_{1}/a$)	orthorhombic P b c a
a (Å)	13.670(3)	13.9026(9)	11.4137(2)	10.1332(2)	10.042(3)
b(Å)	8.9220(2)	8.9784(4)	11.46100(10)	7.62820(10)	7.591(2)
c(Å)	11.890(3)	11.8318(9)	10.7848(2)	27.2405(2)	28.174(3)
β ( ${}^{\circ }$)	111.73(3)	111.418(8)	117.359(2)	90	90
Volume (Å${}^{3}$)	1347.1(5)	1374.89(17)	1252.982 (4)	2105.64(5)	2147.8(9)
Z, Calculated density (Mg/m${}^{3}$)	4, 1.355	4, 1.542	4, 0.154	8, 0.1831	8, 1.387
Absorption coefficient (mm${}^{-1}$)	0.278	2.984	0.126	0.150	0.110
F(000)	576	648	544	880	944
Theta range for data collection (${}^{\circ }$)	2.935 −28.804	2.927 −26.999	3.86 −76.29	3.24 −75.38	3.441 −28.867
Index ranges	−17 ≤ h ≤ 18, −11 ≤ k ≤ 12, −16 ≤ l ≤ 9	−17≤ h≤ 17, −11 ≤ k ≤ 11, −12 ≤ l ≤ 15	−14 ≤ h ≤ 14, −14 ≤ k ≤ 14, −12 ≤ l ≤ 13	−142 ≤ h≤ 10, −9 ≤ k ≤ 9, −32 ≤ l ≤ 33	−13 ≤ h≤ 13, −8≤ k ≤ 10, −36 ≤ l ≤ 37
Reflections Collected unique observed [I > 2sigma(I)]	9623	9471	17416	13106	14345
	3259	2957	2623	2170	2677
	1811	1923	2483	2017	1951
$R_{int}$	0.0713	0.0959	0.0281	0.0401	0.0501
Completeness to Θ (%)	25.242${}^{\circ }$, 99.1	25.242${}^{\circ }$, 99.2	76.29${}^{\circ }$, 99.0	75.38${}^{\circ }$, 99.0	25.242${}^{\circ }$, 99.8
Refinement method	Full-matrix least-squares on $F^{2}$	Full-matrix least-squares on $F^{2}$	Full-matrix least-squares on $F^{2}$	Full-matrix least-squares on $F^{2}$	Full-matrix least-squares on $F^{2}$
Data/restraints/parameters	3259/0/172	2957/0/172	2623/0/259	2170/0/140	2677/0/149
Goodness-of-fit on $F^{2}$	0.883	1.436	4.030	3.16	1.052
Final R indices [I > 2sigma(I)]	$R_{1}$ = 0.0569	$R_{1}$ = 0.0703	$R_{1}$ = 0.0408	$R_{1}$ = 0.0408	$R_{1}$ = 0.0485
	$wR_{2}$ = 0.1289	$wR_{2}$ = 0.1686	$wR_{2}$ = 0.1184	$wR_{2}$ = 0.0534	$wR_{2}$ = 0.1315
R indices (all data)	$R_{1}$ = 0.1079	$R_{1}$ = 0.1149	$R_{1}$ = 0.0422	$R_{1}$ = 0.0429	$R_{1}$ = 0.0669
	$wR_{2}$ = 0.1433	$wR_{2}$ = 0.2216	$wR_{2}$ = 0.1192	$wR_{2}$ = 0.0540	$wR_{2}$ = 0.1384

## Abbreviations

The following abbreviations are used in this manuscript:

NBS	*N*-bromosuccinimide
NCS	*N*-chlorosuccinimide
CFU	the colony forming unit
MTT	3-(4,5-dimethylthiazol-2-yl)-2,5-diphenyltetrazolium bromide
SD	standart deviations
$IC_{50}$	the half maximal inhibitory concentration
NMR	nuclear magnetic resonance
FTIR	Fourier-transform infrared spectroscopy
HR-MS-MS	high-resolution electrospray ionization mass spectra
TLC	thin layer chromatography
